# Thermal Decomposition of 2- and 4-Iodobenzyl Iodide Yields Fulvenallene and Ethynylcyclopentadienes: A Joint Threshold Photoelectron and Matrix Isolation Spectroscopic Study

**DOI:** 10.1021/acs.jpca.3c04688

**Published:** 2023-09-21

**Authors:** Mayank Saraswat, Adrian Portela-Gonzalez, Ginny Karir, Enrique Mendez-Vega, Wolfram Sander, Patrick Hemberger

**Affiliations:** † Lehrstuhl für Organische Chemie II, Ruhr-Universität Bochum, 44780 Bochum, Germany; ‡ Laboratory for Synchrotron Radiation and Femtochemistry, Paul Scherrer Institut (PSI), CH-5232 Villigen, Switzerland

## Abstract

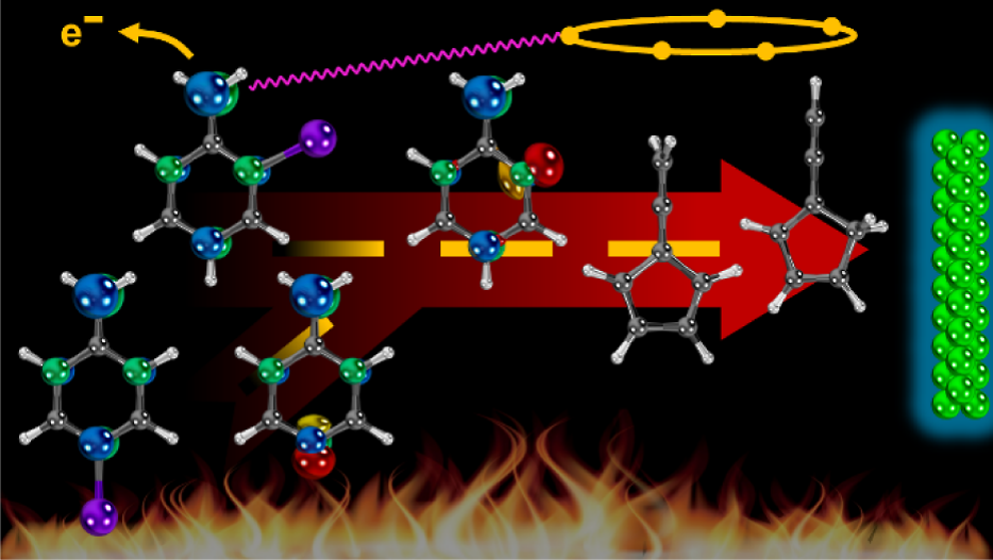

The thermal decomposition of 2- and 4-iodobenzyl iodide at high temperatures was investigated by mass-selective threshold photoelectron spectroscopy (ms-TPES) in the gas phase, as well as by matrix isolation infrared spectroscopy in cryogenic matrices. Scission of the benzylic C–I bond in the precursors at 850 K affords 2- and 4-iodobenzyl radicals (*ortho*- and *para*-IC_6_H_4_CH_2_^•^), respectively, in high yields. The adiabatic ionization energies of *ortho*-IC_6_H_4_CH_2_^•^ to the X̃^+^(^1^A′) and ã^+^(^3^A′) cation states were determined to be 7.31 ± 0.01 and 8.78 ± 0.01 eV, whereas those of *para*-IC_6_H_4_CH_2_^•^ were measured to be 7.17 ± 0.01 eV for X̃^+^(^1^A_1_) and 8.98 ± 0.01 eV for ã^+^(^3^A_1_). Vibrational frequencies of the ring breathing mode were measured to be 560 ± 80 and 240 ± 80 cm^–1^ for the X̃^+^(^1^A′) and ã^+^(^3^A′) cation states of *ortho*-IC_6_H_4_CH_2_^•^, respectively. At higher temperatures, subsequent aryl C–I cleavage takes place to form α,2- and α,4-didehydrotoluene diradicals, which rapidly undergo ring contraction to a stable product, fulvenallene. Nevertheless, the most intense vibrational bands of the elusive α,2- and α,4-didehydrotoluene diradicals were observed in the Ar matrices. In addition, high-energy and astrochemically relevant C_7_H_6_ isomers 1-, 2-, and 5-ethynylcyclopentadiene are observed at even higher pyrolysis temperatures along with fulvenallene. Complementary quantum chemical computations on the C_7_H_6_ potential energy surface predict a feasible reaction cascade at high temperatures from the diradicals to fulvenallene, supporting the experimental observations in both the gas phase and cryogenic matrices.

## Introduction

Astronomical detections, supported by laboratory spectroscopic measurements, have confirmed the presence of a wide variety of molecular species like neutral molecules, ions, and radicals in the interstellar medium (ISM).^1^ Just over the past couple of decades, multiple complex organic molecules (defined as molecules with more than 5 atoms), both saturated and unsaturated ones, have been detected in the ISM.^2,3^ More recently, polycyclic aromatic hydrocarbons (PAHs), containing multiple five- and six-membered fused aromatic rings, have been discovered in low-temperature interstellar environments.^4^ While it has been postulated that PAHs and their ions are possible carriers of unidentified infrared bands in the ISM, understanding PAH chemistry is a topic of great interest among the astronomical community. The simplest aromatic molecule, benzene, and other substituted phenyl derivatives are thought to be the backbone for PAH formation via radical–radical, neutral–radical, and ion–neutral reactions.^5–9^ Toluene **1** (C_7_H_8_) is a methyl-substituted benzene which has also been detected in Titan’s upper atmosphere by the Cassini Ion and Neutral Mass Spectrometer.^10^

Hydrogen detachment from **1** leads to benzyl radical **2** (C_7_H_7_^•^), a key intermediate in combustion processes^11^ and atmospheric chemistry.^12^ Benzyl radical **2** is an aromatic π-type radical with C_2v_ symmetry and a ^2^B_2_ electronic ground state, which has been extensively studied in the gas phase^13–15^ and inert gas matrices,^15–17^ as well as theoretically.^18,19^ Photoionization of radical **2** affords benzyl cation **2**^**+**^, which has been detected in the gas phase using mass-selective threshold photoelectron (ms-TPE) spectroscopy^20,21^ and is also trapped in cryogenic matrices.^22,23^ The adiabatic ionization energies (AIEs) of radical **2** to the corresponding cation **2**^**+**^ in its lowest-energy singlet X̃^+^(^1^A_1_) and triplet spin state ã^+^(^3^B_2_) were recently determined to be 7.25 and 9.18 eV, respectively, leading to a singlet–triplet gap (Δ*E*_S–T_) of 1.93 eV.^20^ The electronic structure of these two states is completely different; the ground-state closed-shell singlet ^1^A_1_ bears a positive charge fully delocalized among the CH_2_ group and the ring due to extended π conjugation, whereas the triplet ^3^B_2_ resembles a diradical where one electron is localized at the CH_2_ group and the other unpaired electron (and the positive charge) is localized on the ring.

**Scheme 1:**
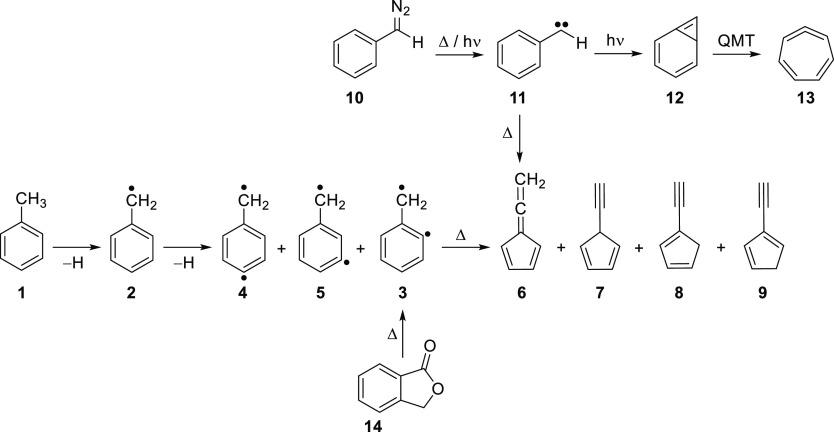
Generation and Isomerization of C_7_H_6_ Species

Further H-loss from radical **2** yields α,*n*-didehydrotoluene **3–5**, which are diradicals bearing a resonance-stabilized benzyl π radical as well as an additional σ radical moiety localized at the ring (Scheme 1). The (anti)ferromagnetic coupling of the unpaired electrons and, hence, the ground-state multiplicity of these diradicals can be rationalized using the disjoint/nondisjoint approach from Borden and Davidson.^24^ Multiconfigurational calculations predict robust triplet ground states for α,2- and α,4-didehydrotoluene **3** and **4**, whereas an open-shell singlet (osS), with a small Δ*E*_S–T_ of about −3 kcal/mol, was estimated for α,3-didehydrotoluene **5**.^25,26^ Open-shell singlet **5** was thermally generated in the gas phase and characterized by photoelectron spectroscopy.^27^ The electronic structure of triplet α,2- and α,4-didehydrotoluene **3** and **4** was later demonstrated by EPR spectroscopy in cryogenic matrices.^28^ While both diradicals **3** and **4** were generated via UV photolysis of suitable precursors, only diradical **4** was detected in matrices. The authors indicate that diradical **3** might rearrange at high temperatures to other more stable closed-shell singlet C_7_H_6_ isomers that could not be identified since they are EPR-silent species.

The C_7_H_6_ potential energy surface (PES) is very rich, containing many experimentally observed open- and closed-shell molecules.^29,30^ Several five-membered C_7_H_6_ isomers like fulvenallene **6**, the lowest-energy isomer, as well as 1-, 2-, and 5-ethynylcyclopentadiene (**8**, **9**, and **7**) have been detected in the Taurus Molecular Cloud (TMC-1).^31,32^ Allene **6** can also be produced in the gas phase by pyrolysis of phenyldiazomethane **10** via formation and in situ ring contraction of the very reactive phenylcarbene **11**.^33^ In contrast, visible-light photolysis of carbene **11** yields the strained bicyclo[4.1.0]heptatriene **12**, which is very unstable and spontaneously rearranges via heavy-atom quantum mechanical tunneling to cycloheptatetraene **13** even at temperatures as low as 3 K.^30^ Alternatively, allene **6** is also produced by thermal decomposition of phthalide (1-isobenzofuranone) **14**, presumably involving the elusive diradical **3**, although such species could not be identified (Scheme 1).^34–36^ In addition, isomers **7–9** as well as a fulvenallenyl radical (via H-loss) were detected and characterized by ms-TPE spectroscopy in the gas phase. In this study, AIEs of 8.23, 8.27, 8.49, and 8.76 ± 0.01 eV were determined for isomers **6**, **8**, **9**, and **7**, respectively.^36^ Moreover, it was pointed out that the C_7_H_6_ isomer distribution inside the hot reactor is not under thermal equilibrium, since irreversible H-detachment competes with isomerization. In a subsequent study, the thermal reaction of *o*-benzyne with a methyl radical at 1130 K was reported to yield benzyl radical **2** (C_7_H_7_), which upon H-loss affords isomers **6–9** (C_7_H_6_); nevertheless, diradicals **3–5** (C_7_H_6_) were not detected.^6^

**Scheme 2:**
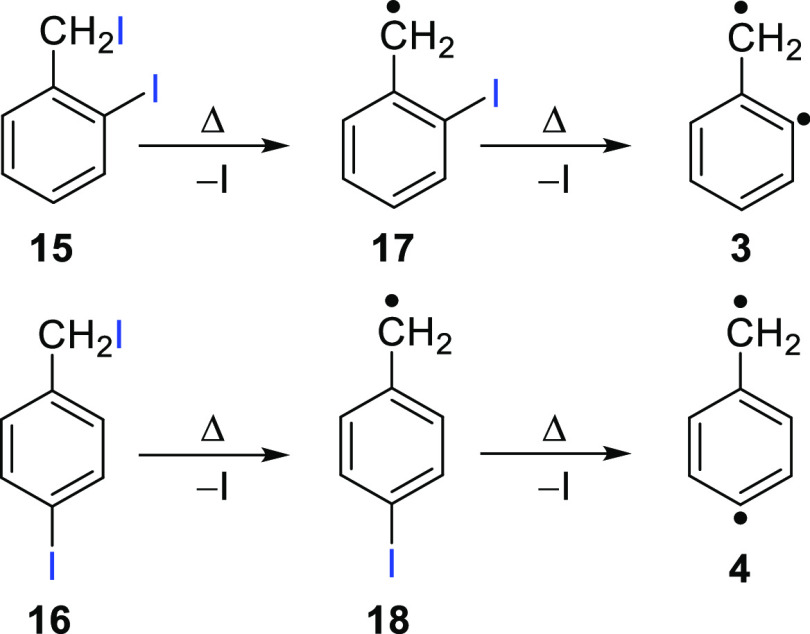
Pyrolytic Route to Diradicals **3** and **4**

In this work, we study the thermal generation of diradicals **3** and **4** and explore their reactivity and isomerization pathways along the C_7_H_6_ PES under pyrolytic conditions by means of ms-TPE spectroscopy in the gas phase as well as by matrix isolation infrared (IR) spectroscopy in cryogenic matrices. Flash vacuum pyrolysis (FVP) of precursors 2-iodobenzyl iodide **15** and 4-iodobenzyl iodide **16** is performed under controlled pyrolysis conditions to selectively produce 2-iodobenzyl radical **17** and 4-iodobenzyl radical **18**, respectively, via loss of an iodine atom. The radicals were fully characterized by gas-phase ms-TPE and low-temperature IR spectroscopy (Scheme 2). Under high-temperature pyrolysis conditions, the second C–I is cleaved to produce several C_7_H_6_ isomers of astrochemical relevance.

## Methods

Precursors **15** and **16** were synthesized from 2- and 4-bromobenzyl iodide, respectively, following a procedure reported in the literature.^28^ Samples **15** and **16** were heated at 50–55 °C and sublimed along with a He flow of 30–40 sccm, and the gas mixture subsequently expanded through a 200 μm nozzle into a pyrolysis reactor. The reactor consisted of a 40 mm long SiC tube with an internal diameter of 1 mm, which is electrically heated by two electrodes separated by 15 mm to 800–1300 K.^37,38^ The products are then characterized using double imaging photoelectron photoion coincidence (CRF-PEPICO) spectroscopy^39^ at the VUV beamline of the Swiss Light Source (SLS) at the Paul Scherrer Institute, Villigen.^40^ Details of this analytical tool have been reviewed in the literature^41,42^ and are only briefly described here.

Following FVP, the molecular beam (MB) is skimmed (2 mm opening) before entering the ionization chamber. Here, a beam of tunable VUV radiation ionizes the products, yielding photoelectrons and photoions that are extracted in opposite directions via an electric field (218 V/cm). The photoions are analyzed using time-of-flight mass spectrometry (MS) and velocity map imaging (VMI), which enables us to distinguish the MB emanating from the hot FVP reactor from background (BG) signals as well as direct ionization (DI) from dissociative photoionization (DPI).^43^ Photoelectrons are also velocity map-imaged according to their kinetic energy release, and electrons of <10 meV are selected to obtain TPE spectra. The advantage of having both ions and electrons in coincidence allows us to collect photoion ms-TPES.^44^ These are recorded by plotting the TPE signal in coincidence with the ion signal in the time-of-flight window of the *m*/*z* of interest as a function of photon energy. Spectra were corrected for false coincidences, and the hot electron contribution was subtracted using the approach by Sztáray and Baer.^45^ Due to the short residence time (25–50 μs) and low pressure (9–20 mbar) in the hot SiC reactor, adiabatic cooling is only limited, leading to activity of hot and sequence band transitions.^38,46^ By applying ion VMI, the rovibrational cooling can be strongly improved, leading to less spectral congestion.^43^ The determined AIEs reported in this article have an uncertainty of 0.01 eV caused by the selected step-size of 10 meV for the recorded spectra. The PEPICO technique coupled to FVP has been used to detect and spectroscopically characterize many fundamental reactive intermediates, in particular, biradicals, as recently reviewed.^47^

Matrix isolation experiments were performed by standard techniques^48^ using two-staged closed-cycle helium refrigerator systems (4 K). Precursors are sublimed and codeposited along with an excess of Ar at a flow rate of 1 sccm onto a CsI window held at 4 K. In FVP experiments, the gas mixture is passed through a hot quartz tube (8 mm diameter and an 80 mm heating zone) kept at 400–1200 K, followed by trapping and IR spectroscopic characterization of the FVP products in the matrix. IR spectra were recorded with a FTIR spectrometer using a resolution of 0.5 cm^–1^ in the range of 400–4000 cm^–1^.

All quantum chemical computations were performed with the Gaussian 16 package.^49^ Optimized geometries of precursors, transition states, intermediates, and products as well as vibrational frequencies were obtained at the B3LYP-D3/def2-TZVP level of theory including the D3 dispersion correction,^50^ although the functional ωB97XD was also employed for radicals **17** and **18**. Additionally, AIEs for diradicals **3** and **4**, and fulvenallene **6** were computed using the CBS-QB3, CBS-APNO, and G4 composite methods.^51^ Franck–Condon (FC) simulations were performed with Gaussian 16 using geometries and vibrational modes calculated at the B3LYP-D3/def2-TZVP level of theory. The stick spectra were subsequently convoluted with a Gaussian function (full width at half-maximum, fwhm = 25 meV).

## Results and Discussion

### Mass Spectra of FVP Products

**Figure 1:**
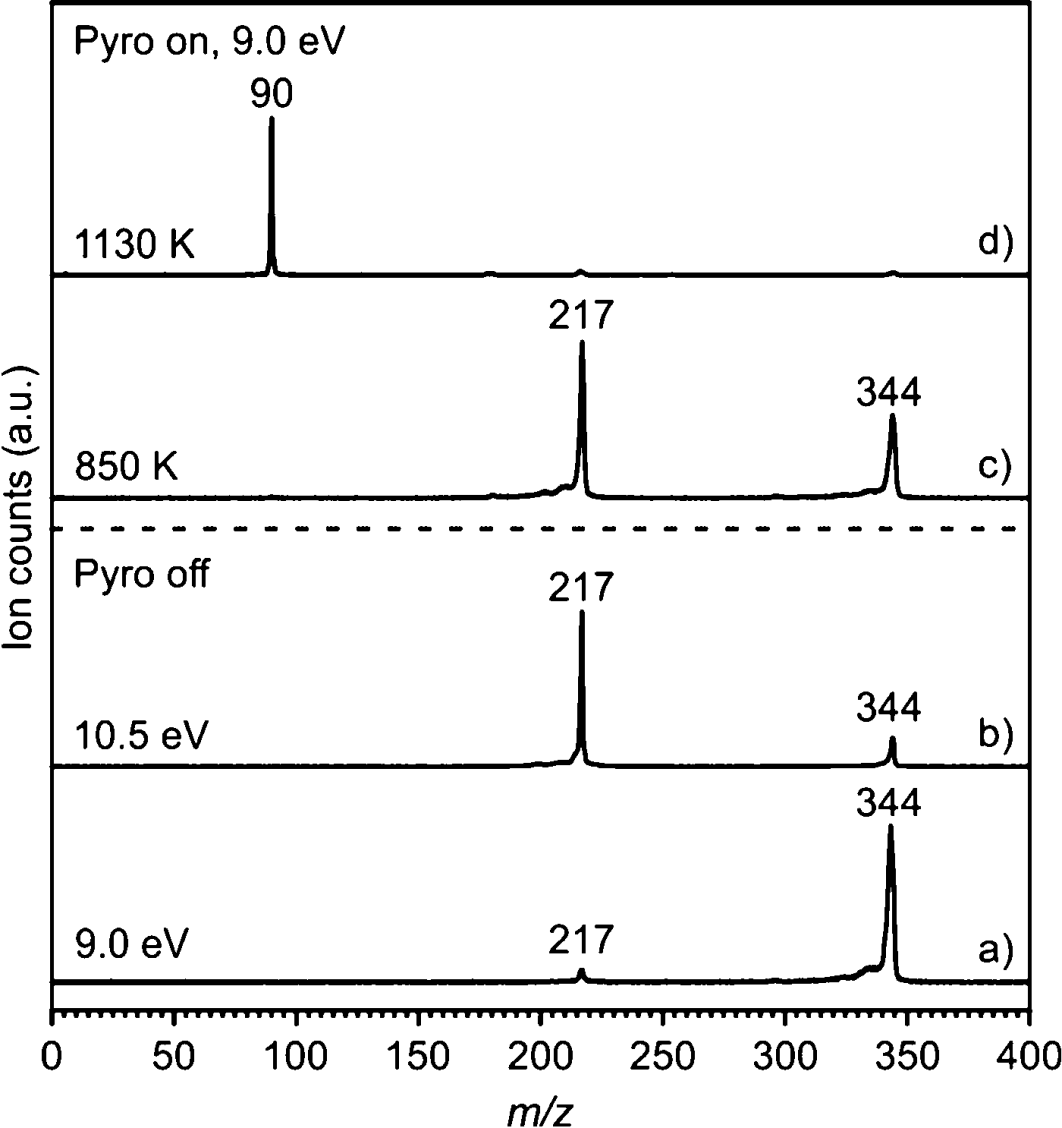
Mass spectra of 2-iodobenzyl iodide **15** recorded at RT (pyrolysis off) with (a) *h*ν = 9.0 eV and (b) *h*ν = 10.5 eV and upon FVP at (c) 850 K and (d) 1130 K.

The thermal decomposition of precursors **15** and **16** (*m*/*z* 344) was studied by photoionization MS as a rapid diagnostic tool at different temperatures and photon energies in the gas phase (Figure 1 and S1 in the Supporting Information). The onset for DI of precursors **15** and **16** (eq 1) at RT (pyrolysis off) appears at 8.50 and 8.35 eV, respectively (Figure S2 in the Supporting Information). However, parent ions **15**^**+**^ and **16**^**+**^ (*m*/*z* 344) are only stable within a narrow photon energy range of ∼0.30 eV, but above that, fragments are formed via DPI (eq 2). The undesired DPI process is also found for benzyl iodide, and the IE is reported to be 8.73 ± 0.02 eV, while the appearance energy of the corresponding radical **2** is 8.84 ± 0.02 eV.^52^ At a fixed photon energy of 9 eV, a very small peak at *m*/*z* 217 is observed, which corresponds to the loss of I (*m*/*z* 127) from precursor **15** (Figure 1a). The peak at *m*/*z* = 217 substantially grows in intensity upon increasing the photon energy to 10.5 eV (Figure 1b). Accordingly, VMI reveals substantial kinetic energy release perpendicular to the MB axis, showing that the fragment ion (*m*/*z* 217) comes from DPI (Figures S3 and S4 in the Supporting Information).
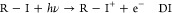
1
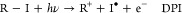
2
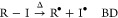
3
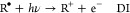
4

FVP of **15** at 850 K results in a substantial depletion of precursor **15** along with the concomitant growth of the peak at *m*/*z* 217 (Figure 1c). Considering that the bond dissociation (BD) energy of the C–I bond in benzyl iodide is 51.1 kcal/mol,^53^ which is substantially lower than that of phenyl iodide (67.7 kcal/mol),^54^ we tentatively assign the peak at *m*/*z* 217 to **17**^**+**^. This ion is mainly formed via DI (eq 4) of the thermally generated radical **17** (eq 3), although DPI of the remaining precursor **15** is also present at 9 eV (Figures S3 and S4 in the Supporting Information). Generally, the onset of DPI is red-shifted for ions with an excess of thermal energy.^43,55^

Upon increasing the pyrolysis temperature to 1130 K, a peak at *m*/*z* 90 is the only peak observed in the spectrum (Figure 1d). In this case, the precursor is completely pyrolyzed, so the ions arise exclusively from DI of the thermal fragments. The peak at *m*/*z* 90 corresponds to the molecular formula C_7_H_6_ and arises from the primary pyrolysis product, diradical **3,** or related isomers **6–9**. Similar results were obtained on the pyrolysis of precursor **16**, although a higher FVP temperature of 1260 K was necessary for the complete depletion of precursor **16**. Identification of all of these pyrolysis products will be conducted in the following sections by means of isomer-specific ms-TPE and matrix isolation IR spectroscopy.

### ms-TPE Spectra of FVP Products

#### 2- and 4-Iodobenzyl Radicals (**17** and **18**, *m*/*z***217**)

ms-TPE spectra are recorded by scanning the photon energy in the range of 7–11 eV and collection of the ms-TPE signal in coincidence with a particular ion. The ms-TPE signal is typically obtained by integrating the whole ion image, comprising a MB and BG. However, in FVP experiments, the MB component exhibits a rovibrational temperature similar to that of the reactor due to expansion from only a few mbar into high vacuum, limiting the adiabatic cooling^38^ and resulting in a broadened ms-TPE spectra with reduced vibrational resolution.^43^ In contrast, as recently shown, the BG in the VMI is populated with molecules rethermalized through wall collisions, which thus benefits from room temperature Boltzmann-like distributions affording ms-TPE spectra with higher resolution. In addition, under mild pyrolysis conditions, the remaining nonpyrolyzed precursor undergoing DPI at photon energies >9 eV adds more complexity to the data analysis of *m*/*z* 217, since hot reactors red shift the onset of dissociative ionization, which leads to spectral congestion.

**Figure 2:**
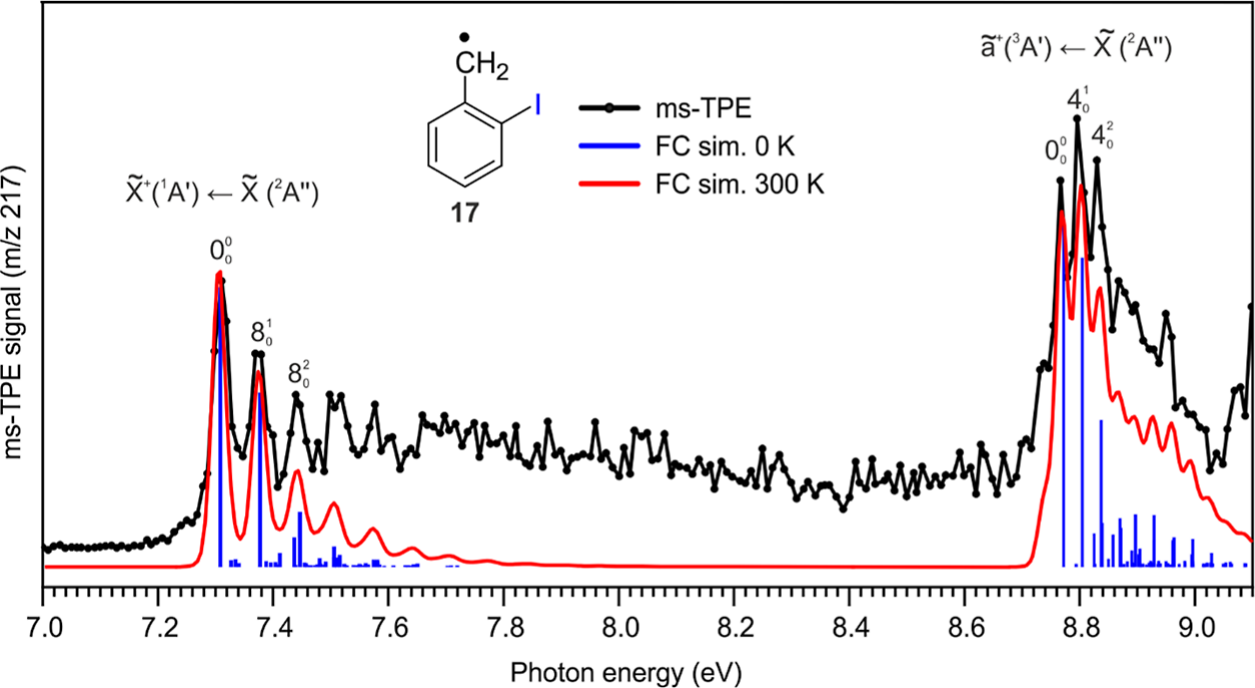
Comparison of the ms-TPE spectrum of *m*/*z* 217 recorded after FVP of 2-iodobenzyl iodide **15** at 850 K (black trace) with FC simulations of 2-iodobenzyl radical **17**. Simulations were performed at 0 K (blue sticks) and at 300 K (red trace) by convolution with 25 meV fwhm Gaussians.

Herein, we report the room-temperature vibrationally cooled ms-TPE spectrum for the signal at *m*/*z* 217 in the range of 7–9.1 eV, which is also corrected for DPI from the nonpyrolyzed precursor. This is accomplished by correcting the rethermalized ions contributing to the BG image in the FVP experiment from the ions formed via DPI in the BG image of the RT (pyrolysis off) experiments (Figure 2 and S5 in the Supporting Information). Since both spectra exclusively contain the room temperature data, a subtraction is possible, and DPI is almost quantitatively suppressed, while vibrational features are revealed. In addition, the difference BG spectrum can be compared to the vibrationally hot MB spectrum, which also suppresses the DPI contributions (Figure S6 in the Supporting Information) by integrating only a narrow region of interest in the MB component of the ion image. This spectrum, however, still suffers from hot and sequence band transitions and thus exhibits a lower vibrational resolution.

The spectrum recorded upon pyrolysis of precursor **15** at 850 K shows two sets of bands with maxima at 7.31 and 8.78 eV (Figure 2). By comparison with the AIEs calculated with different DFT methods, these peaks are assigned to the fundamental (0–0) transition between the zero-point energy (ZPE) levels of radical **17** and the corresponding 2-iodobenzyl cation **17**^**+**^ (with *C*_*s*_ symmetry) in its lowest-energy singlet X̃^+^(^1^A′) and triplet ã^+^(^3^A′) spin states. The difference between the AIEs of **17** and the lowest-energy singlet and triplet states allows us to experimentally determine the Δ*E*_ST_ of cation **17**^**+**^ to be 1.47 eV, which is reasonably reproduced by DFT calculations (Table 1). In addition, a vibrational progression is observed between 7.3 and 7.5 eV with clear peaks at 7.31, 7.38, and 7.45 eV, allowing us to gain vibrational information on cation **S-17**^**+**^. Such progression is dominated by the origin band 0–0 and excitations of the ring breathing mode (ν_8_) with a spacing of 70 ± 10 meV (560 ± 80 cm^–1^), which compares well with the calculated unscaled frequency of singlet **17**^**+**^ at the B3LYP-D3/def2-TZVP level of theory (557 cm^–1^). Similarly, another vibrational progression is observed within 8.7–8.9 eV with a smaller spacing of 30 ± 10 meV (240 ± 80 cm^–1^), which is nicely reproduced by the calculated ν_4_ mode of **T-17**^**+**^ (263 cm^–1^).

**Table 1:**
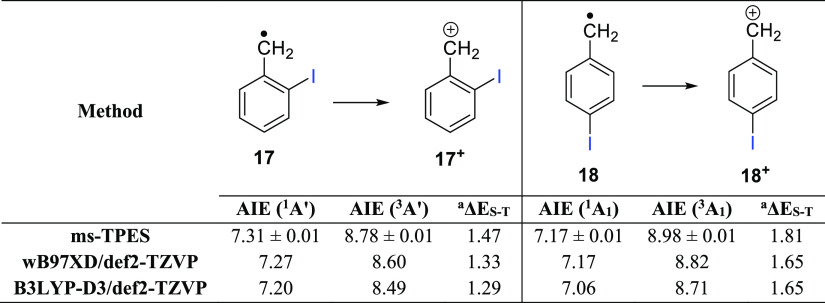
Experimental and Calculated AIEs of 2- and 4-Iodobenzyl Radicals (in eV)

a Singlet–triplet energy splitting (Δ*E*_ST_) of **17**^**+**^ and **18**^**+**^. Energies are shown in eV and are ZPE-corrected.

**Figure 3:**
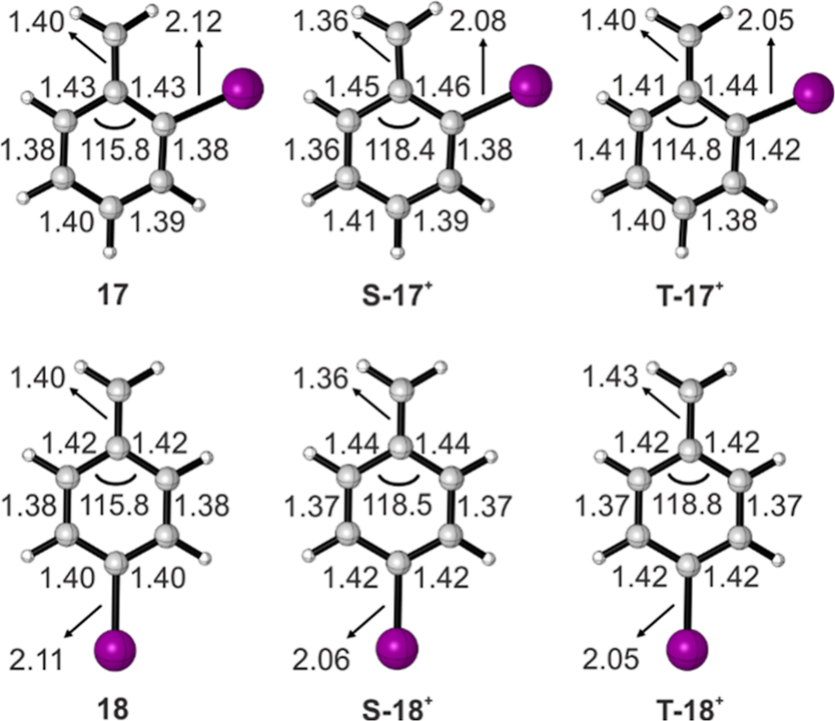
Optimized geometries of 2- and 4-iodobenzyl radicals **17** and **18** and their corresponding cations in singlet and triplet states at the B3LYP-D3/def2-TZVP level of theory. Selected bond distances and angles are shown in Å and degrees, respectively.

The ms-TPE spectrum corresponding to *m*/*z* 217 obtained via FVP of precursor **16** at 850 K exhibits two band systems with maxima at 7.17 and 8.98 eV (Figure S9 in the Supporting Information), which are assigned to the vibronic transition from radical **18** to the corresponding 4-iodobenzyl cation **18**^**+**^ (with *C*_2*v*_ symmetry) in its singlet X̃^+^(^1^A_1_) and triplet ã^+^(^3^A_1_) spin states (Figure 3). Such experimental AIE as well as the Δ*E*_S–T_ of 1.81 eV compares well with the values estimated by DFT (Table 1). Unfortunately, the lower resolution of the TPE spectrum prevents the acquisition of vibrational data on cation **18**^**+**^ (Figures S7–S9 in the Supporting Information).

The ms-TPE spectra allow us to experimentally determine Δ*E*_S–T_ values of 1.47 and 1.81 eV for cations **17**^**+**^ and **18**^**+**^, respectively. As comparison, AIEs of 7.25 and 9.18 eV were measured for the benzyl cation **2**^**+**^, resulting in a Δ*E*_S–T_ of 1.93 eV.^20^ In benzyl radical derivatives (**2**, **17**, and **18**), electron detachment from the singly occupied molecular orbital (SOMO) yields closed-shell singlet states, whereas the triplet states are formed by ionization from the doubly occupied orbitals (HOMO – 1) on the conjugated π system (Figure S10 in the Supporting Information). The absolute energies and nature of the SOMO orbitals are very similar in radicals **2**, **17**, and **18**; hence, according Koopman’s theorem, ionization energies to the singlet states should not differ much, in agreement with the experimental data (Table 1). In contrast, a significant red-shift between 0.2 and 0.4 eV is observed for the analogue transitions to the triplet states of **17**^**+**^ and **18**^**+**^ with respect to that of **2**^**+**^. The HOMO – 1 in radicals **17** and **18** is mainly localized at the I atom, bearing the out-of-plane lone pair, and is relatively higher in energy than the HOMO – 1 in the parent radical **2** (Figure S10 in the Supporting Information). The increase in the energy of the HOMO – 1 orbital is even more noticeable for the ortho isomer **17**, leading to a smaller AIE to the triplet state. Accordingly, the triplet AIE and the Δ*E*_S–T_ follow the order *ortho***17** < *para***18** < parent **2**, matching the experimental data (Table 1). The strong dependency of the Δ*E*_S–T_ on the position of the iodine substituent attached to the ring is in line with the trend observed in the isomeric xylyl (methyl-benzyl) radicals.^56^

The optimized geometries of the singlet and triplet states of cations **17**^**+**^ and **18**^**+**^ were carefully inspected (Figure 3). The C–C bond between the phenyl ring and the CH_2_ group in **S-17**^**+**^ and **S-18**^**+**^ is substantially shorter with respect to that in **T-17**^**+**^ and **T-18**^**+**^ and also to radicals **17** and **18**. This shortening arises from the strong π-electron donation from the ring to the electron-deficient CH_2_ moiety in **S-17**^**+**^ and **S-18**^**+**^, pointing to a detachment of the unpaired π electron (SOMO). In contrast, **T-17**^**+**^ and **T-18**^**+**^ experience a shortening in the C–I bond as compared to radicals **17** and **18**, which suggests that the ionization takes place from the out-of-plane lone pair of iodine (HOMO – 1).

Our approach, including the correction of the ms-TPE by the BG subtraction of the FVP and RT spectra, revealed three high-energy sets of bands with maxima at 9.10, 9.68, and 10.49 eV that are tentatively assigned to excited states of **17**^**+**^ (Figures S6 and S7 in the Supporting Information). Electronic transitions from radical **17** to the Ã^+^(^1^A′) and b̃^+^(^3^A″) excited states of **17**^**+**^ are estimated by TD-ωB97XD/def2-TZVP calculations to be 8.80 and 9.46 eV, which reasonably fit to the experimental values. These electronic states arise from the removal of an electron from the HOMO – 1 and HOMO – 2, respectively, with the open-shell singlet Ã^+^(^1^A′) state being closely related to that of ã^+^(^3^A′).

#### C_7_H_6_ Isomers (*m*/*z***90**)

**Figure 4:**
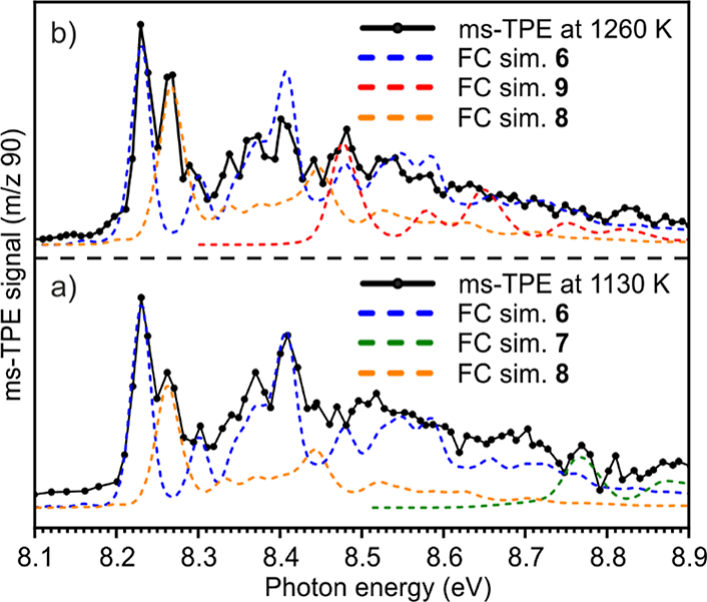
Comparison of the ms-TPE spectrum of *m*/*z* 90 with FC simulations of isomers **6–9**. (a) FVP of precursor **15** at 1130 K and (b) FVP of precursor **16** at 1260 K. Simulations were performed at 1130 and 1260 K by convolution with 25 meV fwhm Gaussians.

ms-TPE spectra were also recorded for the signal at *m*/*z* 90, obtained upon FVP of both precursors **15** and **16** under harsh conditions. FVP of **15** at 1130 K gives characteristic peaks at 8.23 and 8.26 and a small peak at 8.77 eV, which match the AIEs reported for isomers **6**, **8**, and **7**,^36^ respectively (Figure 4a). However, the spectrum could not be fitted by the transitions of the expected primary thermal product, diradical **3,** calculated with several DFT and composite methods (Table S1 in the Supporting Information). Likewise, other high-energy C_7_H_6_ isomers such as **12** and **13** were also ruled out by comparison with the calculated ionization energies. FVP of precursor **16** at 1260 K gives a ms-TPE spectrum (Figure 4b) which partially resembles that obtained by FVP of the isomeric precursor **15**. Nevertheless, the spectrum possesses clear differences such as the intensity pattern, the absence of the signal at 8.77 eV tentatively assigned to **7**, and the appearance of a new peak at 8.49 eV, which fits to the isomer **9** by comparison with literature.^36^

We extended the search for signals at *m*/*z* 90 by examining mass spectra at lower photon energies. Interestingly, a small peak at *m*/*z* 91 appears in the spectrum recorded upon FVP of precursor **16** at 1260 K within the range of 7.25–7.6 eV. This peak can be assigned to the benzyl radical **2** with a reported AIE of 7.25 eV.^20^ This is an indirect indication of the transient presence of diradical **4** in the gas phase, which is readily quenched by H atoms in the pyrolysis reactor besides the rearrangements discussed above (main deactivation channel). An alternative approach to trap reactive intermediates for an extended time span is utilizing FVP in combination with cryogenic matrices. This technique will be discussed in the next section.

#### IR Spectra of FVP Products in Cryogenic Matrices

**Figure 5:**
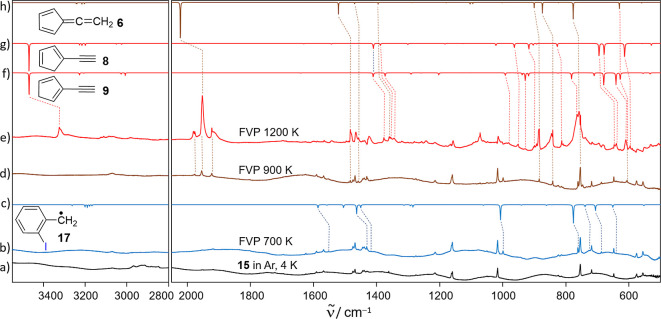
IR spectra of the FVP of 2-iodobenzyl iodide **15** in Ar matrices at 4 K. (a) Deposition of **15** at RT (pyrolysis off). (b) FVP of **15** at 700 K. (c) Calculated IR spectrum of radical **17** at B3LYP-D3/def2-TZVP. (d,e) FVP of **15** at 900 K. (f–h) Calculated IR spectrum of isomers **6**, **8**, and **9** at B3LYP-D3/def2-TZVP.

Matrix isolation experiments in cryogenic matrices were conducted to confirm the identity of the trapped FVP products of precursors **15** and **16** by using IR spectroscopy. The reactor temperature was scanned from RT (pyrolysis off) to 1200 K, and IR spectra were taken similarly to the methodology used in the MS experiments in the gas phase (Figure 5). As mentioned before, we will fully discuss the FVP of precursor **15**, while similar experiments on precursor **16** are shown in Figures S13–S15 in the Supporting Information.

The IR spectrum of matrix-isolated precursor **15** in Ar matrices at 4 K exhibits three characteristic bands at 1160, 1016, and 754 cm^–1^ that are used to monitor the thermal decomposition of the precursor at higher temperatures (Figure 5a). FVP of precursor **15** at 600–700 K results in the appearance of new signals at 1431, 999, 762, and 687 cm^–1^ (Figure 5b and S1 in the Supporting Information), which compare well to the calculated IR spectrum of radical **17** (see full assignment in Table S2 in the Supporting Information). Upon increasing the temperature to 800 K, a new IR signal at 747 cm^–1^ appears which is only visible in the range of 800–900 K. This band fits to the most intense vibration (out-of-plane deformation at 757 cm^–1^) of triplet diradical **3**, calculated at the B3LYP-D3/def2-TZVP level of theory (Figure S12 in the Supporting Information). All other bands of diradical **3** are predicted to be much weaker and are not observable in our spectra.

Further increase of the FVP temperature to 900–1000 K results in the selective formation of **6** with a very intense peak at 1954 cm^–1^, which corresponds to a characteristic allene vibration. Moreover, the IR spectrum fits to that reported in the literature^57^ and also to the calculated IR spectrum. However, above this temperature, a mixture of ethynylcyclopentadienes **8** and **9** is observed with characteristic vibrations at ∼3325 cm^–1^ based on comparison with their reported^57^ and calculated IR spectra. In contrast to ms-TPES, high-energy isomer **7** was not observed in the matrix, presumably due to the longer residence time of the species inside the hot reactor in the matrix isolation setup.

Similarly, in the case of the isomeric precursor **16**, FVP at 600–700 K yields the corresponding radical **18** in agreement with DFT calculations (see full assignment in Figure S13 and Table S3 in the Supporting Information). Upon further increase of the pyrolysis temperature, product **6** as well as alkynes **8** and **9** are observed. Interestingly, we also detect a band at 793 cm^–1^ under mild pyrolysis conditions (800–900 K), which is tentatively assigned to triplet diradical **4** (Figure S14 in the Supporting Information). This band is analogous to that observed for triplet diradical **3** and matches the most intense vibration (out-of-plane deformation at 810 cm^–1^) calculated at the B3LYP-D3/def2-TZVP level of theory (Figure S12 in the Supporting Information). The triplet diradicals **3** and **4** are formed only as traces detected by IR spectroscopy.

A similar product distribution is obtained from the thermal decomposition of either precursor **15** or **16** in the gas phase and in cryogenic matrices if comparable pyrolysis conditions are used (Figure S15 in the Supporting Information). Hence, several questions arise such as (a) why are diradicals **3** and **4** only transient species in the gas phase, (b) do they interconvert, and (c) how are they transformed into allene **6**. We will discuss these points in the following section by computationally exploring the PES and rationalizing the experimental observations obtained in the gas and solid phases.

#### Thermal Rearrangements of Didehydrotoluene Diradicals

**Figure 6:**
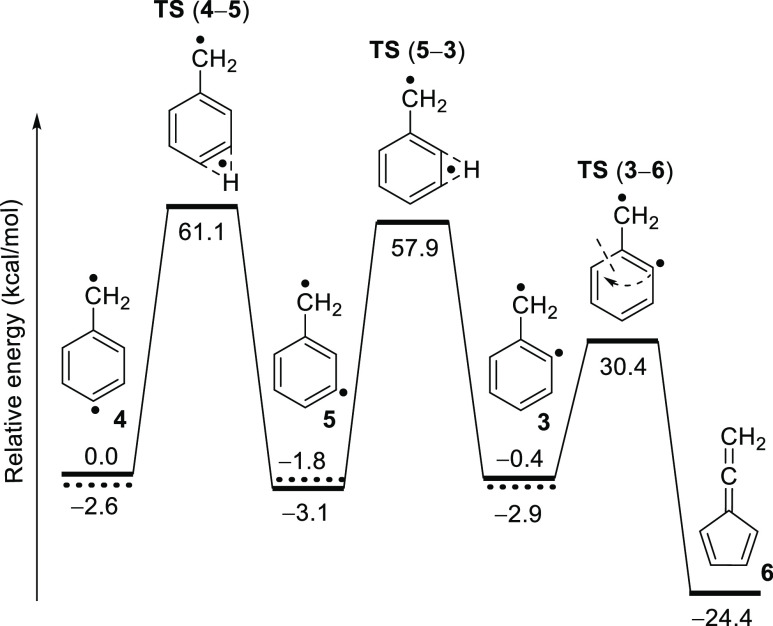
PES (singlet) for thermal rearrangement of diradicals **3** and **4** to fulvenallene **6** calculated at the ZPE-corrected B3LYP-D3/def2-TZVP level of theory. Triplet states for diradicals **3**–**5** shown as dashed levels.

Diradicals **3** and **4** are experimentally generated via homolytic cleavage of C–I bonds from the corresponding radicals **17** and **18** under pyrolytic conditions (Scheme 2). This means that they are initially formed in vibrationally excited states that undergo vibrational relaxation to the corresponding open-shell singlet states, which can either further decay via intersystem crossing to their triplet ground states or rearrange to lower-energy isomers on the singlet PES. Herein, we computationally explore the landscape corresponding to the thermal rearrangement of diradicals **3** and **4** to allene **6** (Figure 6). The closed-shell singlet states of diradicals **3–5** and their connecting TSs are found to lie much higher in energy and should not be involved (Figure S16 in the Supporting Information).

Allene **6** is the global minimum (or thermodynamic sink) of the C_7_H_6_ PES, being >20 kcal/mol more stable than diradicals **3–5** in either their lowest-energy open-shell singlet or triplet states. The ring contraction of diradical **3** to allene **6** is estimated to proceed via a concerted mechanism, overcoming a barrier of 30.8 kcal/mol. In contrast, a direct H-shift connecting diradical **4** and allene **6** is symmetry-forbidden and, hence, must take place by three consecutive steps (**4** → **5** → **3** → **6**), involving two consecutive H-shifts and a final ring contraction. Since every subsequent barrier is smaller than the previous one, a reaction cascade is expected to occur from diradical **4** to allene **6**.

**Scheme 3:**
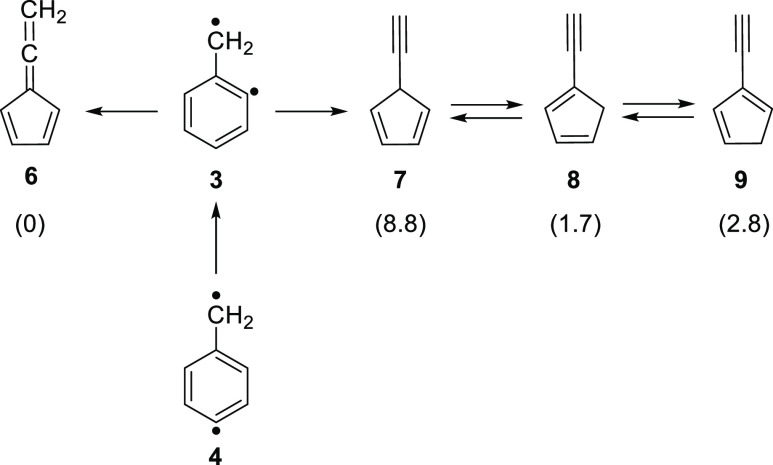
Thermal Ring Contraction of Diradical **3** Relative free energies (Δ*G*, in kcal/mol) of isomers **6–9** calculated with G4.

As discussed before, a mixture of isomeric alkynes is detected in the gas phase at very high temperatures (Figure 4 and Scheme 3). However, the product distribution and relative concentration of these species depend on the pyrolysis temperature. At 1130 K, the kinetic products (initially formed), allene **6** and the high-energy alkyne **7**, are observed along with isomer **8**. In contrast, at 1260 K, isomer **7** is absent and, instead, isomer **9** is now detected. Under these harsh conditions, all barriers (**7** → **8** → **9**) are overcome, affording thermodynamic products. Accordingly, the ratio between isomers **6**:**8**:**9**:**7** is predicted by G4 calculations at 1260 K to be 1:0.51:0.32:0.03, matching the experimental observations (Figure 4, upper panel). The selectivity observed is similar to the thermal product distribution reported by Bouwman et al.^36^

## Conclusions

Disentangling chemical reactions in extreme environments such as the ISM and combustion flames is challenging due to the presence of high-energy radicals, diradicals, ions, and their excited states. These elusive species can be observed on the fly by very sensitive spectroscopic techniques or by trapping at low temperatures. In this study, we combine two tools to elucidate the thermal decomposition of 2- and 4-iodobenzyl iodide **15** and **16** by means of ms-TPES spectroscopy in the gas phase as well as by matrix isolation IR spectroscopy in cryogenic matrices.

FVP of precursors **15** and **16** provide high yields of the corresponding 2- and 4-iodobenzyl radicals **17** and **18**, respectively, allowing the electronic and vibrational spectroscopic characterization of these iodo-substituted benzyl derivatives for the first time. The AIEs of radical **17** to its cation in its lowest-energy singlet X̃^+^(^1^A′) and triplet ã^+^(^3^A′) spin states were determined to be 7.31 ± 0.01 and 8.78 ± 0.01 eV, whereas those of radical **18** were measured to be 7.17 ± 0.01 eV for X̃^+^(^1^A_1_) and 8.98 ± 0.01 eV for ã^+^(^3^A_1_). The ms-TPE spectra allow experimental determination of the singlet–triplet energy gap (Δ*E*_S–T_) of 1.47 and 1.81 eV for cations **17**^**+**^ and **18**^**+**^, respectively. These Δ*E*_S–T_ values are smaller than that of the parent benzyl cation **2**^**+**^ (Δ*E*_S–T_ = 1.93 eV)^20^ due to the destabilization of the HOMO – 1 bearing the out-of-plane lone pair from iodine and, thus, lower AIEs to the triplet states. Moreover, vibrational frequencies of ring breathing modes were measured at 560 ± 80 and 240 ± 80 cm^–1^ for the singlet and triplet states, respectively, of cation **17**^**+**^. Such rarely available spectroscopic information on excited states is valuable for unveiling their geometric and electronic structures as well as for benchmarking theoretical methods.

At higher temperatures, a subsequent loss of the second I atom occurs, producing the reactive diradicals **3** and **4**, which so far could only be characterized by EPR spectroscopy in cryogenic matrices. In argon matrices, diradicals **3** and **4** show IR signals at 747 and 793 cm^–1^, respectively, which are assigned to the out-of-plane deformations computed at 757 and 810 cm^–1^ by DFT. However, the assignment of diradicals **3** and **4** in the matrix is tentative due to the low intensity of the corresponding IR bands. These radicals could not be detected in the gas phase using photoionization MS since they readily rearrange to more stable closed-shell isomers.

Finally, we shed light on thermal rearrangements in the C_7_H_6_ PES, which has been of relevance to astrochemistry since the detection of five-membered ring C_7_H_6_ isomers in the TMC-1. Highly energetic conditions trigger a reaction cascade starting from diradicals **3** and **4** that involve two consecutive H-shifts (**4** → **5** → **3**) followed by ring contraction (**3** → **6**) to form allene **6**. This reaction sequence explains the higher reactivity of diradical **3** compared to diradical **4** toward ring contraction, in agreement with experimental observations.

## Supporting Information Available

Mass spectra of FVP of 4-iodobenzyl iodide **16**, photoionization of precursors **15** and **16**, VMI results, ms-TPE spectra of 2- and 4-iodobenzyl radicals (**17** and **18**), molecular orbitals of 2- and 4-iodobenzyl radicals (**17** and **18**), relative energies and AIEs of C_7_H_6_ isomers, matrix isolation IR spectroscopy, PES of C_7_H_6_, and optimized geometries (PDF)
