# Supplementary material for: Thermal Decomposition of 2- and 4-Iodobenzyl Iodide Yields Fulvenallene and Ethynylcyclopentadienes: A Joint Threshold Photoelectron and Matrix Isolation Spectroscopic Study
Source: J Phys Chem A. 2023 Sep 21;127(41):8574–83. doi: 10.1021/acs.jpca.3c04688 (PMC10591508; doi:10.1021/acs.jpca.3c04688)
Supplement: Supplementary file 1 — jp3c04688_si_001.pdf [file jp3c04688_si_001.pdf]

Thermal Decomposition of 2- and 4-Iodobenzyl Iodide Yields Fulvenallene and Ethynylcyclopentadienes: A Joint Threshold Photoelectron and Matrix Isolation Spectroscopic Study.

Mayank Saraswat[1§], Adrian Portela-Gonzalez[1§], Ginny Karir[1], Enrique Mendez-Vega[1], Wolfram Sander[1*], and Patrick Hemberger[2*]

[1]Lehrstuhl für Organische Chemie II, Ruhr Universität Bochum, 44780 Bochum, Germany
[2]Laboratory for Synchrotron Radiation and Femtochemistry, Paul Scherrer Institut, CH-5232 Villigen, Switzerland

Table of Contents

Mass Spectra of FVP of 4-Iodobenzyl Iodide 16 .... S2
Photoionization of Precursors 15 and 16 .... S3
Velocity Map Imaging S4
ms-TPE Spectra of 2- and 4-Iodobenzyl Radicals (17 and 18) S6
Molecular Orbitals of 2- and 4-Iodobenzyl Radicals (17 and 18) S10
Relative Energies and AIEs of C_7H_6 Isomers S11
Matrix Isolation IR Spectroscopy S12
Potential Energy Surface of C_7H_6 .... S16
Optimized Geometries S17
References S19

Mass Spectra of FVP of 4-Iodobenzyl Iodide 16

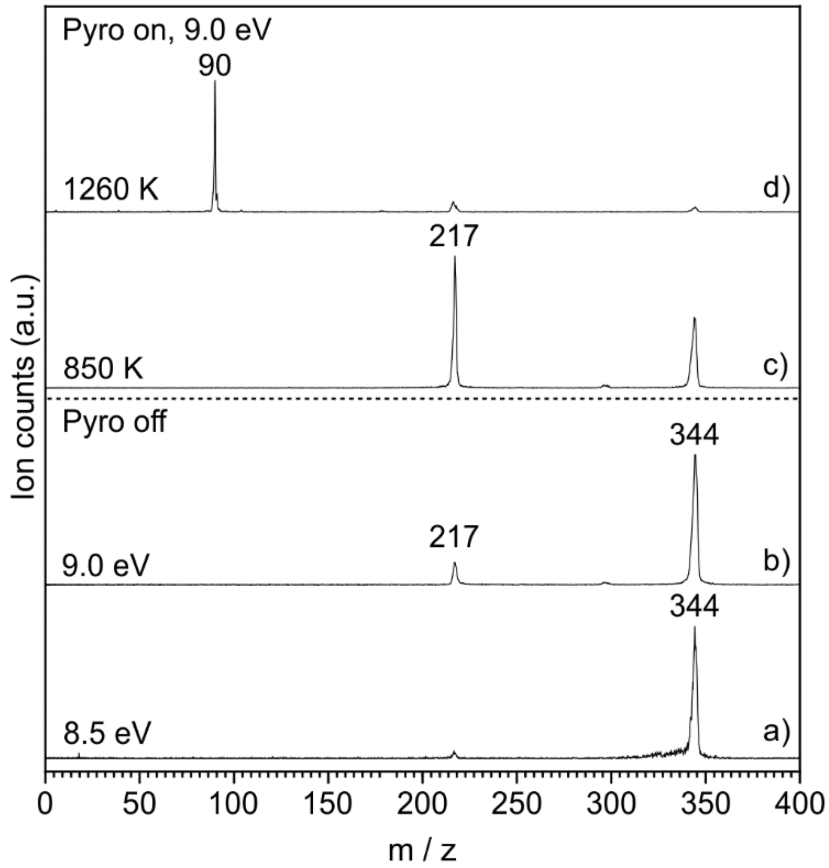

Figure S1. Mass spectrum of 4-iodobenzyl iodide **16** at RT recorded at a photon energy of 8.5 eV (a) and 9.0 eV (b). Mass spectrum of the FVP of **16** at 850 K (c) and 1260 K (d), recorded at a photon energy of 9.0 eV.

Photoionization of Precursors 15 and 16

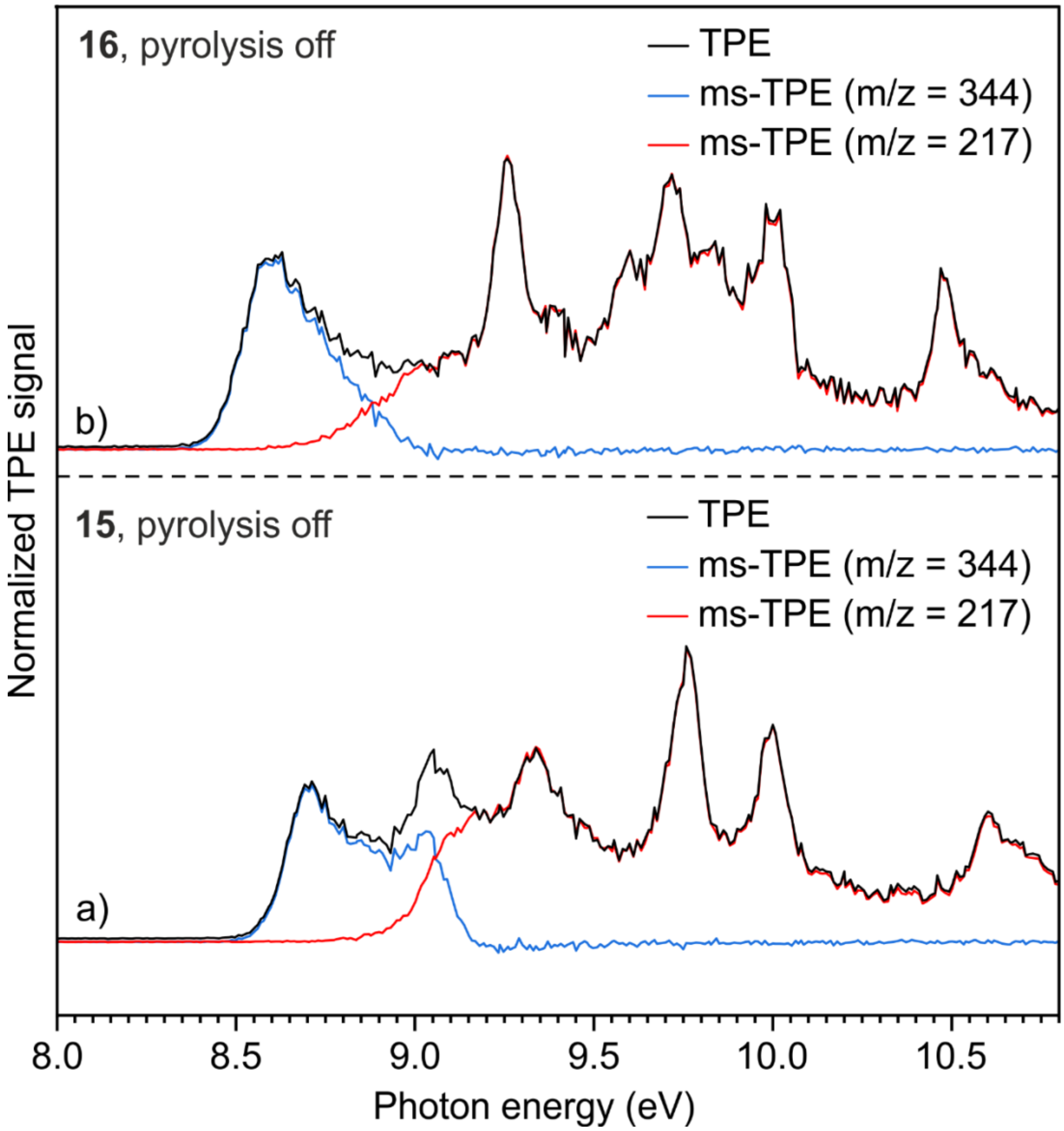

Figure S2. Comparison of the normalized TPE (all ions) and ms-TPE spectra of the precursor (m/z = 344) and its fragment (m/z = 217) of **15** (a) and **16** (b) at room temperature. The onset of the direct ionization of precursor **15** starts at 8.50 eV, whereas its DPI starts at 8.85 eV. The onset of the direct ionization of precursor **16** starts at 8.35 eV and its DPI at 8.65 eV.

Velocity Map Imaging

In velocity map imaging (VMI), ions are focused onto concentric rings according to their momentum in the image plane. The ion image is centered in the background (BG), corresponding to molecules that are rethermalized upon collisions with the wall, and hence exhibit a room temperature (RT) velocity distribution. In the absence of external factors, the x and y momentum components of the BG should be similar, so it appears as a circle in the center of the ion image. On the other hand, the molecular beam (MB) possesses a high velocity due to the adiabatic expansion into vacuum and is thus offset from the BG. Due to skimming the MB exhibits a narrow velocity distribution perpendicular to the molecular beam expansion direction (Figures S3–4). In the VMI image (Figure S3, left), the yellow-red narrow line shows a large concentration of ions with a velocity distribution in the molecular beam (MB) part of the ion image indicative for direct ionization (DI) of radical **17** produced by FVP in the SiC reactor. Those molecules exhibit a ms-TPES of radical **17** at nearly the temperature of the reactor, thus showing low resolution (see below).[1] However some kinetic energy release is also present in the MB part of the ion image, which is a sign of dissociative ionization, especially when compared to the 298 K data (Figure S3, right). On the other hand, the background (BG, in blue) at m/z = 217 contains the information of the molecules that are rethermalized upon collisions with the wall and diffuse back into the ionization region of the PEPICO spectrometer.[1] This includes radical **17** if it is stable enough to survive the wall collisions but vibrationally cooled to (nearly) room temperature as well as fragments formed upon DPI of the unpyrolyzed precursor **15**. Integration of the different areas (MB or BG) leads to ms-TPE spectra differentiating DI from DPI processes, that will be discussed in the next section. On the right part of Figure S3 we observe the ion image of m/z = 217 with pyrolysis off, which shows a broad velocity distribution of ions in the molecular beam, clearly indicating that those ions are purely formed upon DPI with large translational access energies.

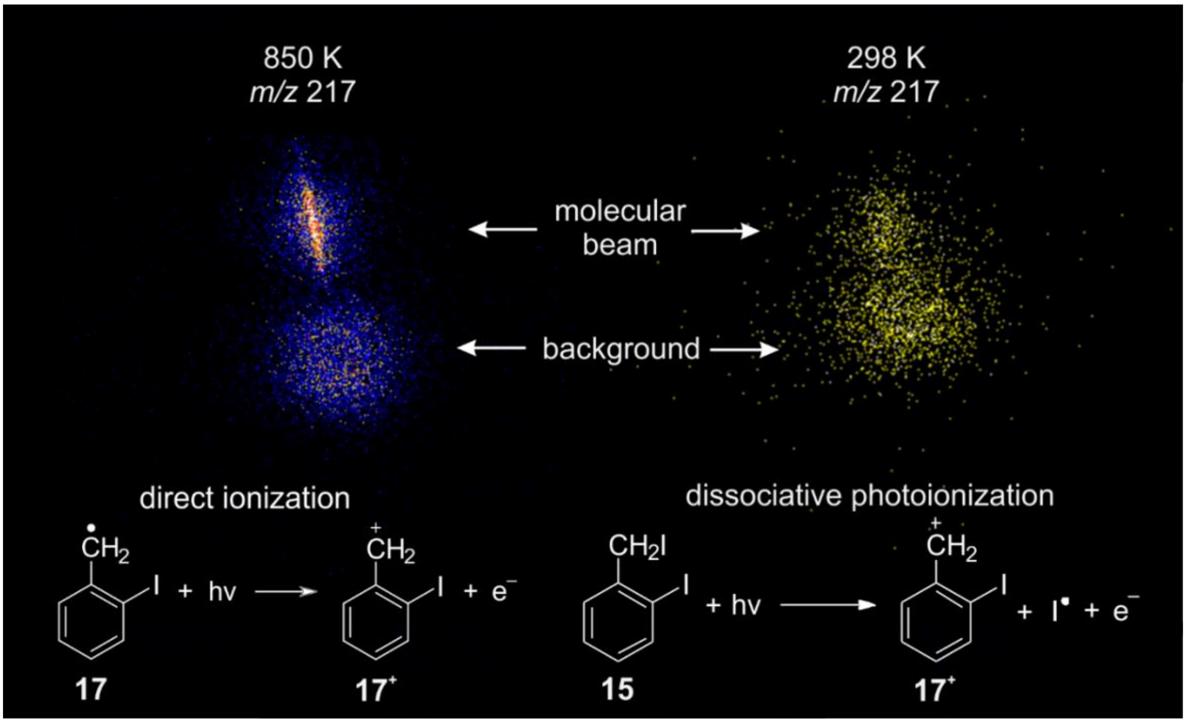

Figure S3. VMI image showing the direct ionization of radical **17** (left) and dissociative photoionization of precursor **15** (left) leading to cation **17**$^+$ at a photon energy of 9.0 eV.

The same behavior was observed for precursor **16** in correspondence with its mass spectrum (Figure S1) and VMI images at 850 K and RT (Figure S4). In this case, the signal corresponding to DPI is even larger than that for isomer **15** (Figure S3).

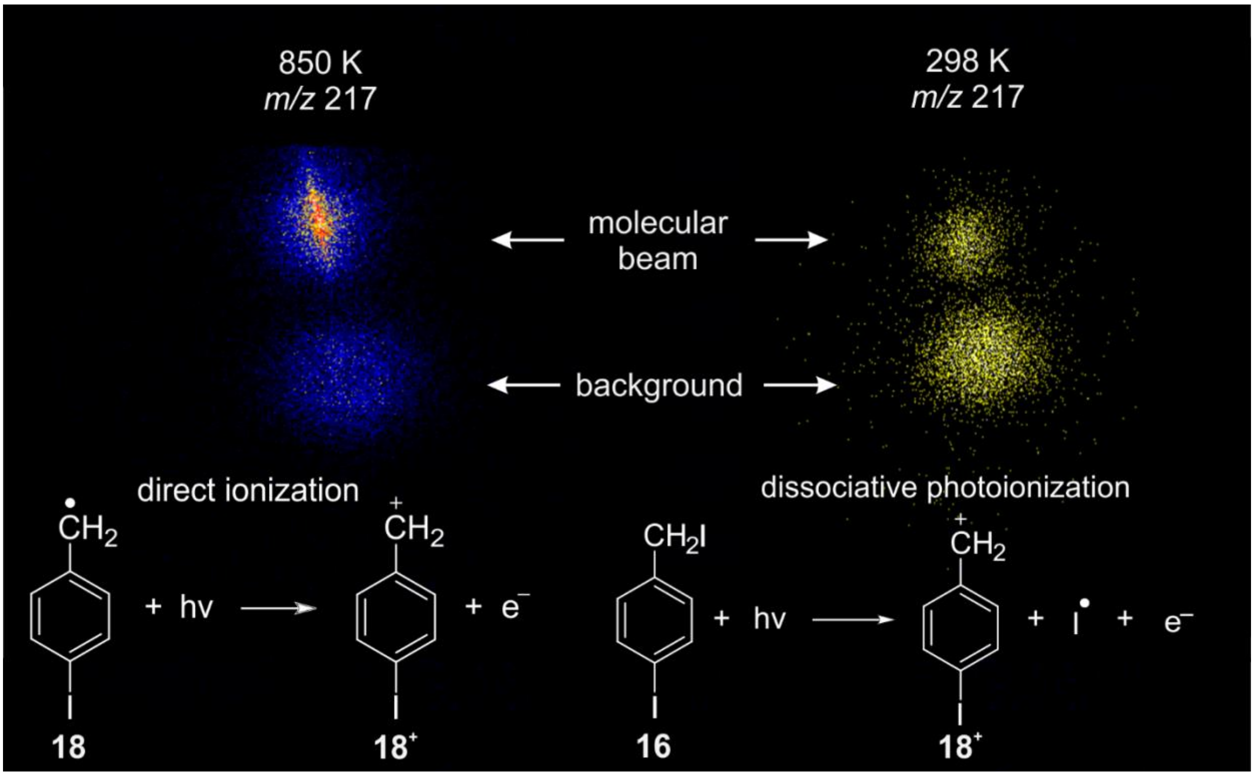

Figure S4. VMI image showing the direct ionization of radical **18** (left) and dissociative photoionization of precursor **16** (left) leading to cation **18**$^+$ at a photon energy of 9.0 eV.

ms-TPE Spectra of 2- and 4-Iodobenzyl Radicals (17 and 18)

Vibrationally-cooled ms-TPE spectrum of 2-iodobenzyl radical 17

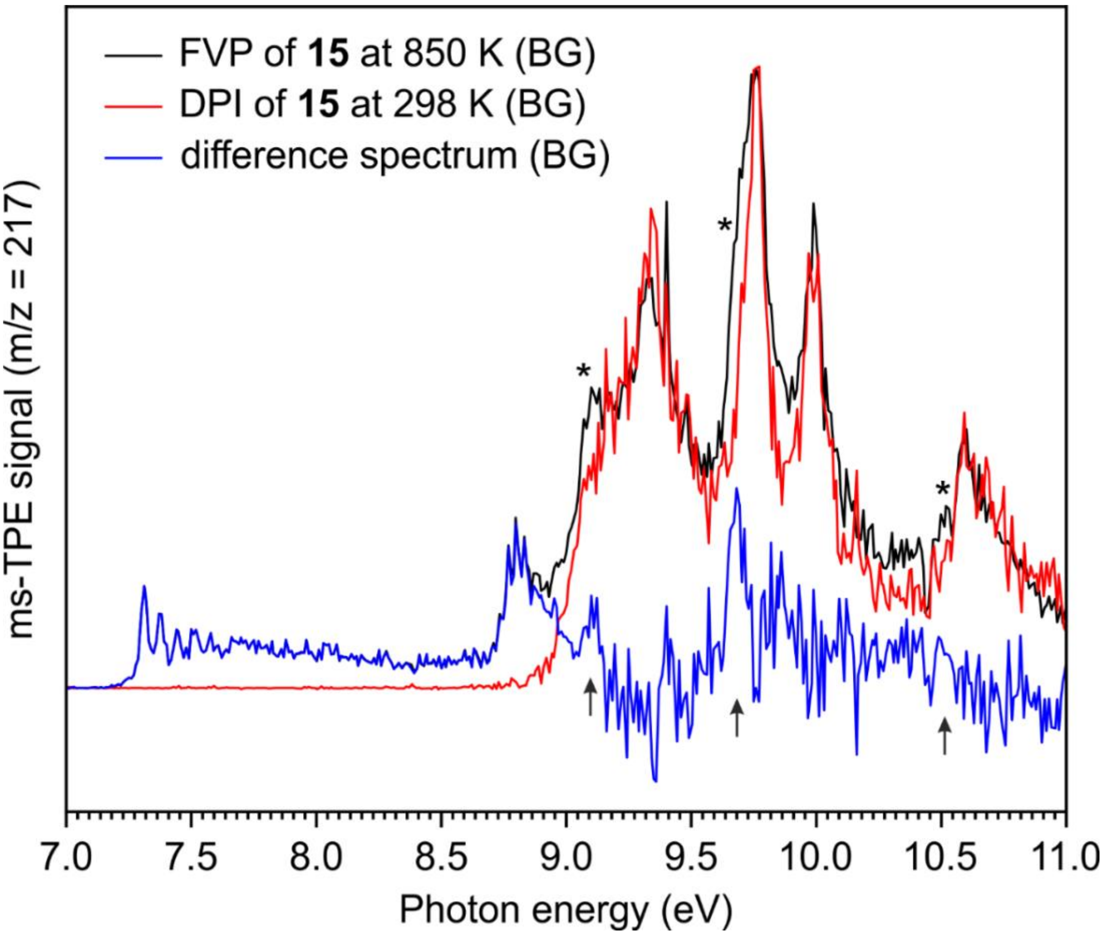

Figure S5. ms-TPE spectra comparing the signal (m/z = 217) obtained by integrating the rethermalized background obtained upon FVP of **15** at 850 K (black trace) and DPI of **15** at 298 K (red trace). The difference spectrum between both spectra is defined as an improved spectrum (shown in Figure 2 of the manuscript) without the undesired contribution of the DPI process and hot bands (blue trace). The arrows and stars indicate the three regions of the difference spectra that show bands assigned to excited states.

Since precursor **15** is not fully pyrolyzed at 850 K, remaining **15** undergoes DPI resulting in very broad and intense bands at photon energies higher than 8.85 eV (Figure S5, black trace). For this reason, we attempted to remove the contribution of DPI processes by recording a reference spectrum of precursor **15** at RT (Figure S5, red trace). The background VMI data, containing the rethermalized molecules, was selected as the region of interest (ROI) for both spectra so they have comparable temperatures. Moreover, the BG spectrum benefits from higher resolution after rethermalization of the molecules thanks to wall collisions.[1] The resulting vibrationally-cooled difference spectrum (Figure S5, blue trace) shows an improved triplet band system (8.7–9.0 eV) with better vibrational resolution, allowing to assign the vibrational progression (see Figure 2 of the manuscript). Moreover,

above 9 eV, three sets of peaks with maxima at 9.10, 9.68 and 10.49 eV are now observable and are assigned to higher-energy excited states of **17**$^{+}$.

The difference BG spectrum (Figure S6, blue trace) was also compared to the spectrum obtained upon selecting the whole ion image (WI, Figure S6, black trace) as well as to the vibrationally hot molecular beam (MB, Figure S6, red trace). The spectrum obtained from the whole ion image exhibits a poor resolution and is hampered by hot and sequence band transitions (red-shifted), which are not satisfactorily reproduced by FC simulations even when set to the pyrolysis temperature. In the high-energy region above 9 eV, only a broad feature centered at 9.63 eV could be identified, which reasonably fits to the band at 9.68 eV observed in the difference BG spectrum assigned to excited states of **17**$^{+}$. However, the other two high-energy transitions are not clearly observable in the MB spectrum.

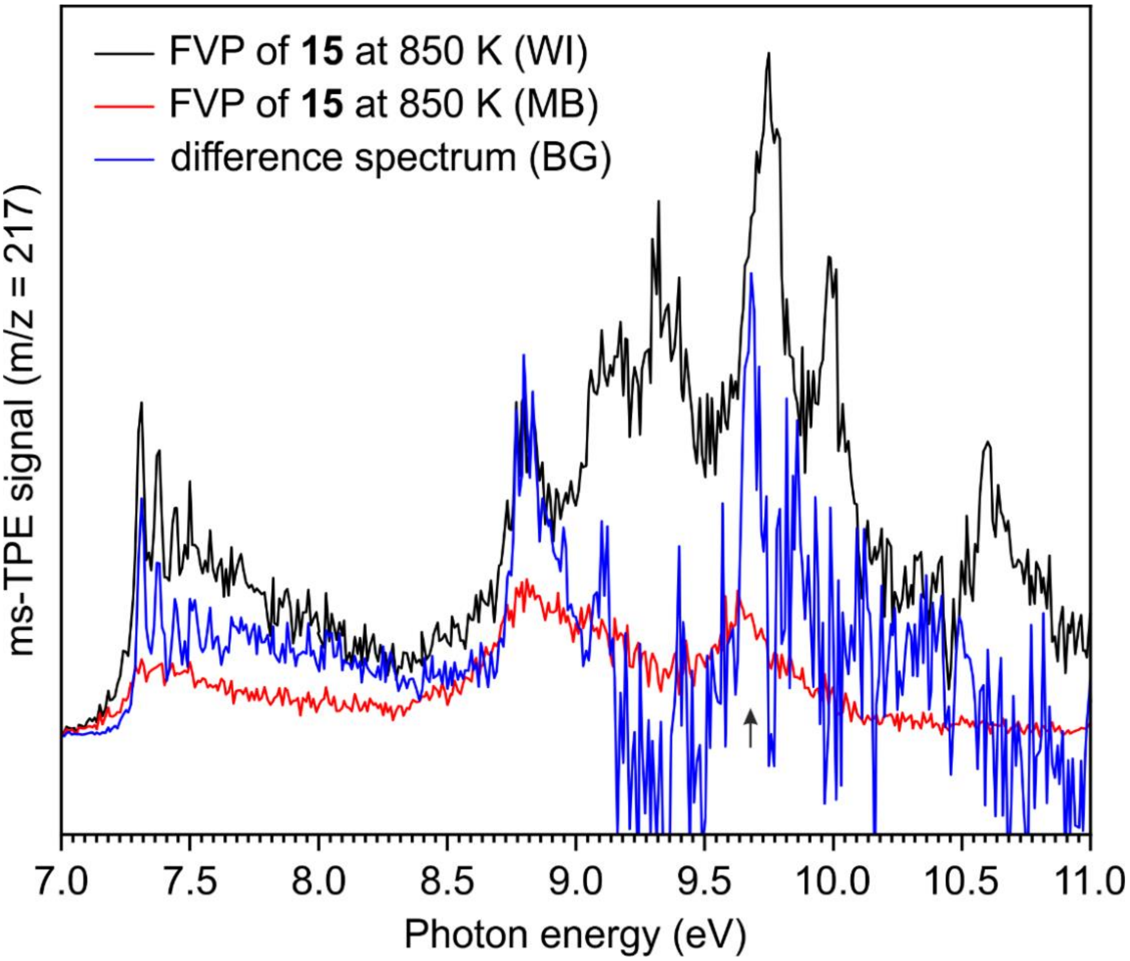

Figure S6. ms-TPE spectra comparing the signal (m/z = 217) obtained by integrating the whole ion image (WI, black trace), the hot molecular beam (MB, red trace) and the difference BG spectrum (blue trace) shown in Figure S4. The arrow indicates a band at 9.63 eV that is tentatively assigned to an excited state.

Vibrationally-cooled ms-TPE spectrum of 4-iodobenzyl radical 18

The same approach was also used for obtaining a vibrationally-cooled ms-TPE spectrum of 4-iodobenzyl radical **18**. In this case, the DPI process starts at a lower photon energy (8.70 eV, Figure S7, red trace). This fact, together with the blue shift of the triplet transition cause a more severe overlap of that band. The benefit of the background correction in this case is even more pronounced than for radical **17**. However, the lower signal-to-noise ratio prevents the observations of high-energy transitions that might correspond to excited states.

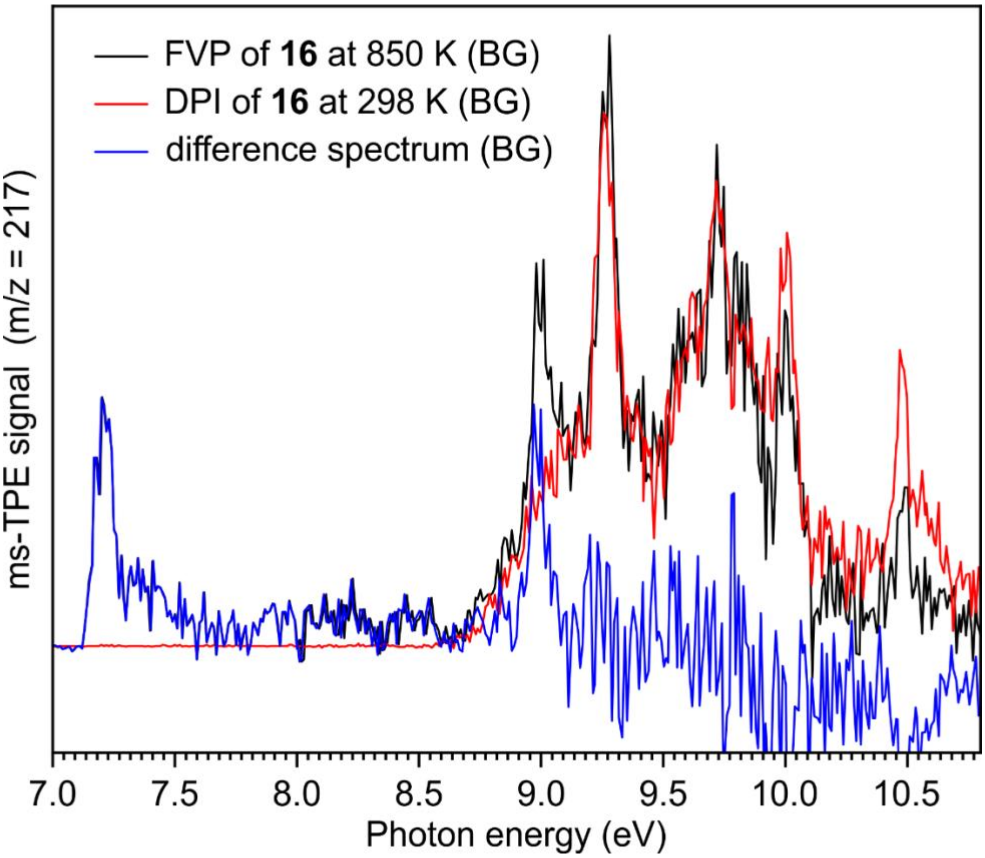

Figure S7. ms-TPE spectra comparing the signal (m/z = 217) obtained by integrating the rethermalized background obtained upon FVP of **16** at 850 K (black trace) and DPI of **16** at 298 K (red trace). Their difference BG spectrum is shown in blue.

The comparison of the difference BG spectrum with that of the whole ion image also shows improvements in the resolution of the singlet and triplet bands and a lower influence of the hot bands (Figure S8). Due to the low intensity of the signal, the spectrum of the MB was not very revealing, and the signals were close to the noise level. Since no higher-energy bands could be observed in the difference BG spectrum, its comparison with the MB spectrum is less relevant.

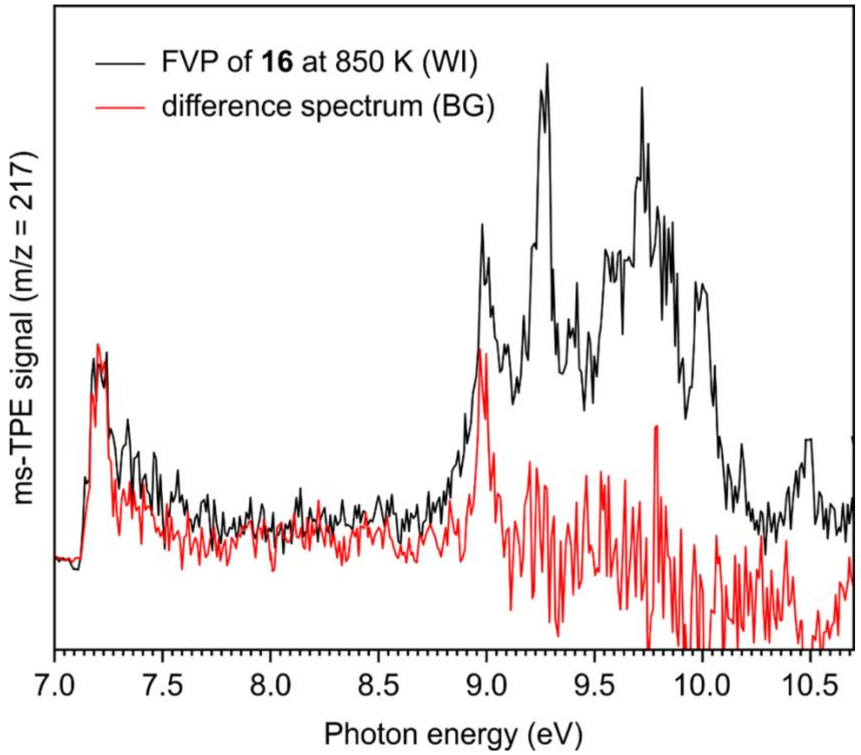

Figure S8. ms-TPE spectra comparing the signal (m/z = 217) obtained by integrating the whole ion image (WI, black trace) with the difference BG spectrum (red trace).

The difference BG spectrum of radical **18** (Figure S9, black trace) was fitted by Franck-Condon simulations at 0 K (blue bars) and 300 K (red trace, broadened with fwhm of 25 meV). The spectrum is split into the singlet and triplet band regions for clarity.

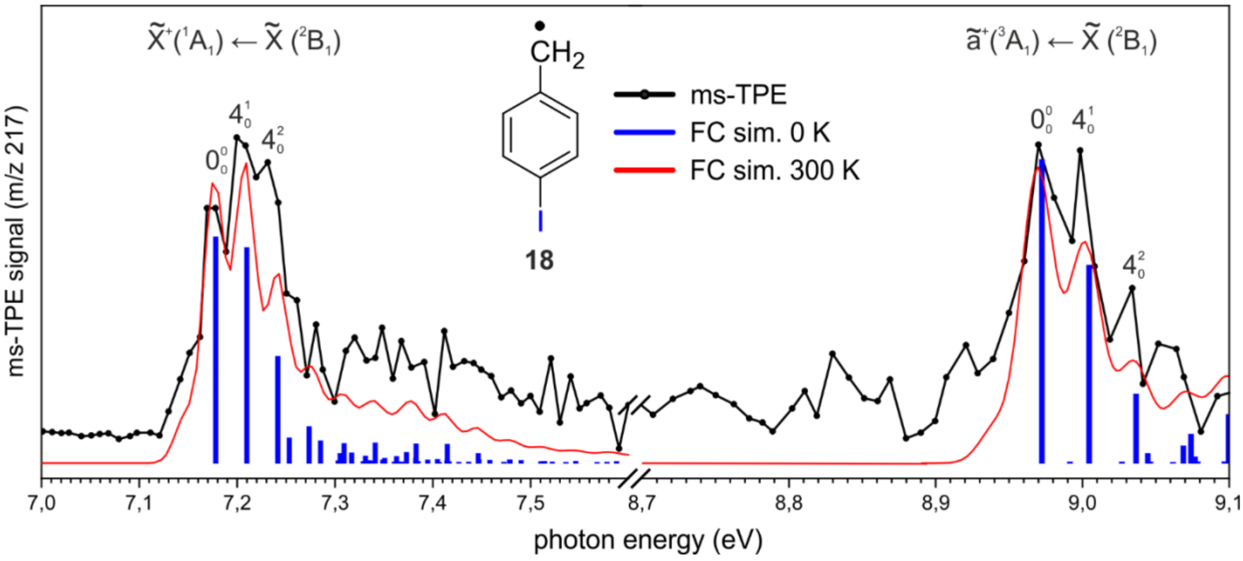

Figure S9. ms-TPE spectrum of m/z = 217 obtained upon FVP of precursor **16** at 850 K (black trace). This spectrum was obtained with the same approach as Figure 2, subtracting the background spectrum obtained from FVP at 850 K and DPI at 298 K of precursor **16**. Simulations were performed at 0 K (blue sticks), and at 300 K (red trace) by convolution with 25 meV fwhm Gaussians.

Molecular Orbitals of 2- and 4-Iodobenzyl Radicals (17 and 18)

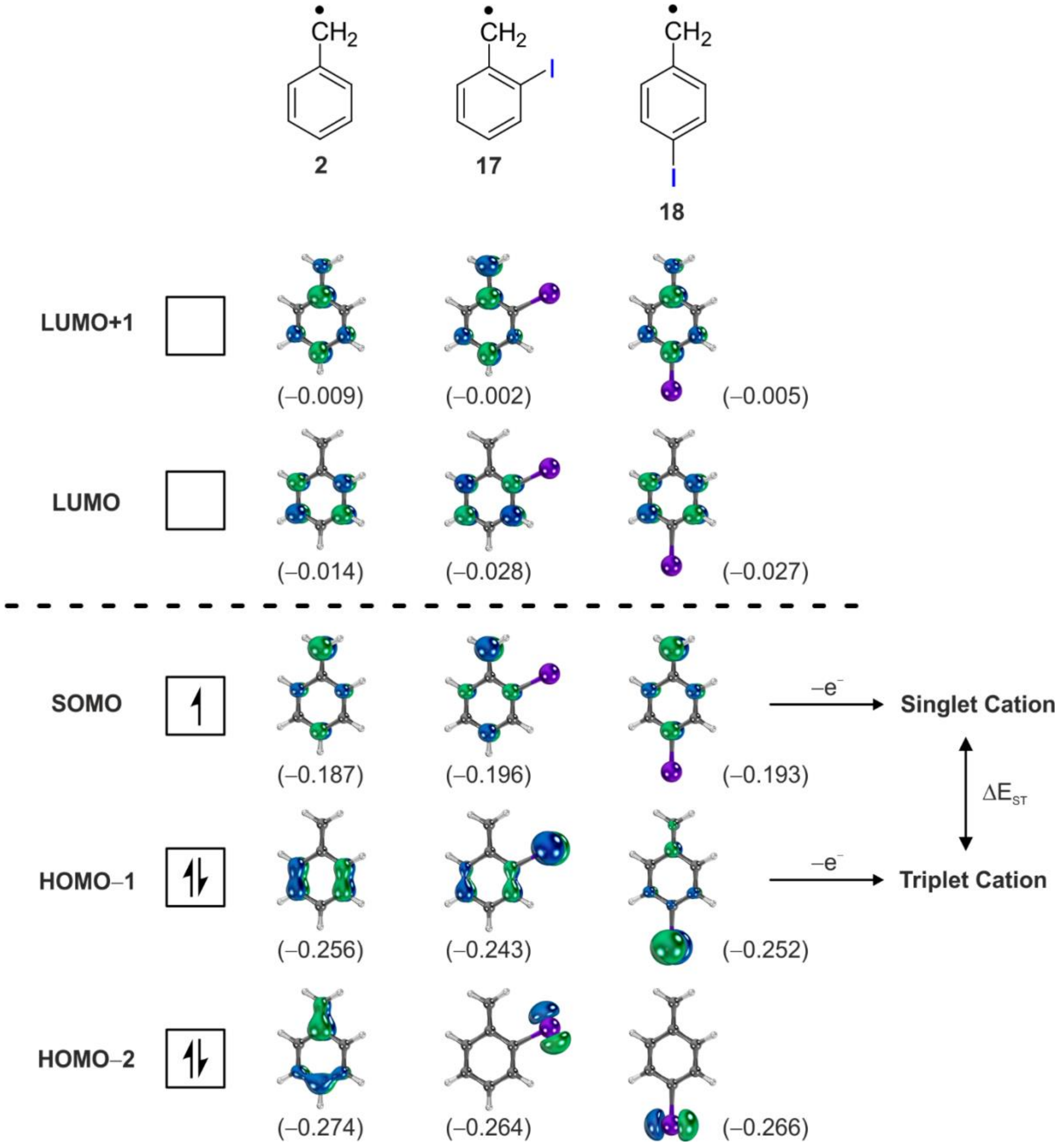

Figure S10. Depiction of selected molecular orbitals (MOs) of 2- and 4-iodobenzyl radicals **17** and **18** as well as of the parent benzyl radical **2**. Absolute energies of the MOs given in Hartrees, and calculated at the B3LYP-D3/def2-TZVP level of theory.

Relative Energies and AIEs of C_7H_6 Isomers

Table S1. Relative energies (in kcal/mol) and adiabatic ionization energies (in eV) of C_7H_6 isomers at different levels of theory.

method	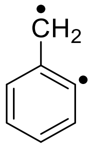 **3**	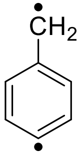 **4**	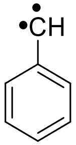	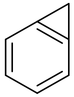	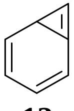 **12**	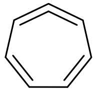 **13**	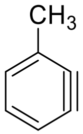	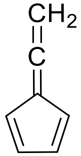 **6**	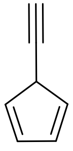 **7**	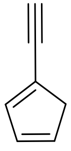 **8**	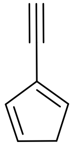 **9**
B3LYP-D3/ def2-TZVP	**21.5** (7.37)	**21.7** (7.29)	**21.5** (7.35)	**7.3** (8.55)	**28.7** (7.80)	**13.0** (7.16)	**20.5** (8.88)	**0.0** (7.91)	**14.2** (8.31)	**4.6** (7.87)	**6.4** (8.08)
CBS-QB3	**25.4** (7.47)	**25.4** (7.42)	**24.6** (7.47)	**5.3** (8.86)	**25.4** (8.08)	**11.8** (7.63)	**15.8** (9.22)	**0.0** (8.26)	**9.6** (8.71)	**2.3** (8.25)	**3.4** (8.49)
CBS-APNO	**25.3** (7.48)	**25.3** (7.42)	**24.3** (7.50)	**4.8** (8.86)	**25.6** (7.45)	**11.8** (7.58)	**15.3** (9.17)	**0.0** (8.24)	**9.5** (8.69)	**1.8** (8.23)	**2.9** (8.46)
G4	**26.5** (7.44)	**26.5** (7.37)	**25.1** (7.48)	**6.8** (8.80)	**25.5** (8.15)	**12.0** (7.60)	**16.1** (9.18)	**0.0** (8.23)	**9.3** (8.70)	**2.2** (8.28)	**3.2** (8.49)

Bold: Relative energies (in kcal/mol); and parenthesis: adiabatic ionization energies (in eV)

Matrix Isolation IR Spectroscopy

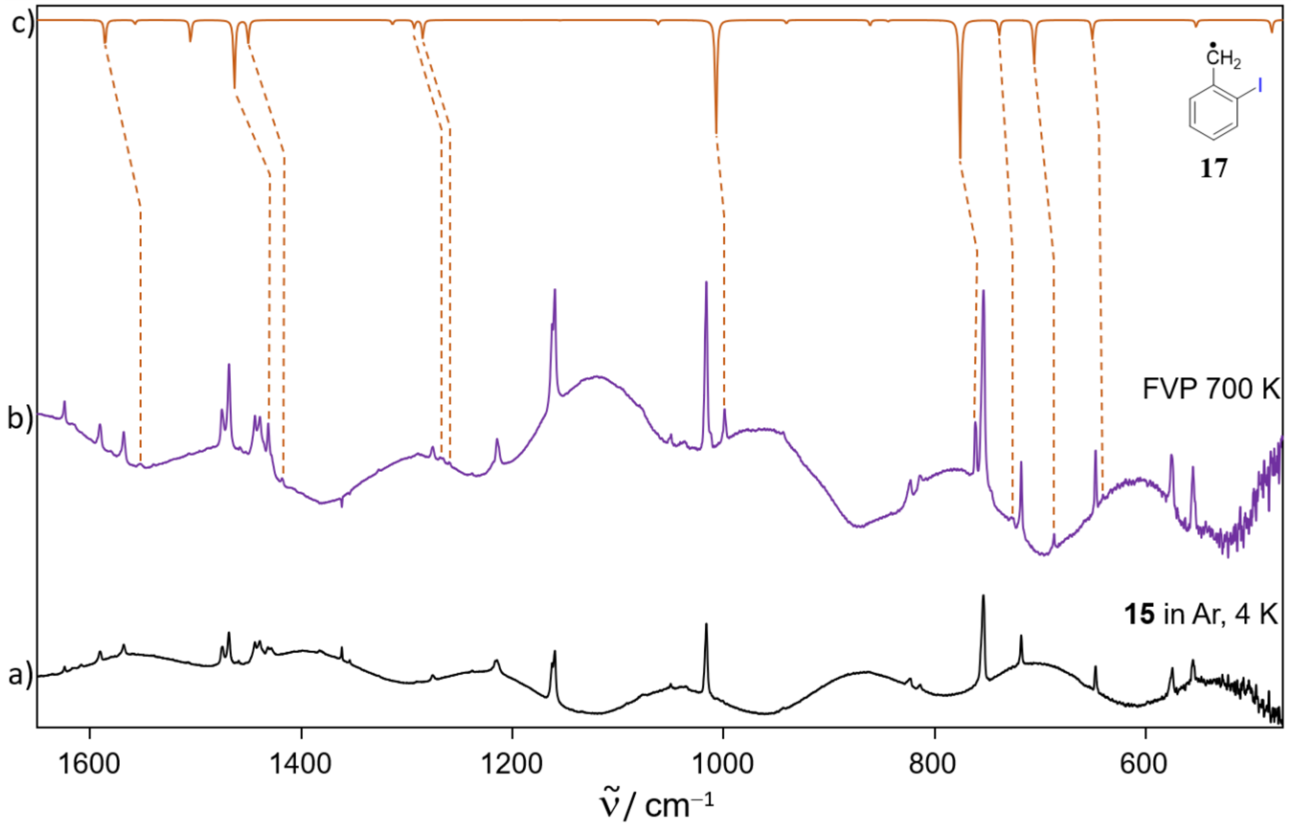

Figure S11. IR spectra of the FVP of 2-iodobenzyl iodide **15** in Ar matrices at 4 K. a) Deposition of **15** at RT (pyrolysis off). b) FVP of **15** at 700 K c) Calculated IR spectrum of radical **17** at the B3LYP-D3/def2-TZVP level of theory.

Table S2. Experimental and calculated IR spectroscopic data of 2-iodobenzyl radical 17.

Argon[a]		Calculated[b]				Assignment
ṽ (cm^{-1})	$I_{rel.}$[d]	normal mode	ṽ (cm^{-1})	$I_{abs.}$[c] (km/mol)	$I_{rel.}$[d]	
641	0.20	10	651	8	0.13	C–C–C bending
687	0.34	11	706	20	0.31	C–H wagging
726	0.14	12	739	7	0.11	C–H wagging
762	1.00	13	776	64	1.00	C–H wagging
999	0.67	19	1007	53	0.82	C–C–C bending
1260	0.10	23	1285	8	0.12	C–H bending
1266	0.11	24	1293	4	0.05	C–H bending
1418	0.19	26	1450	10	0.16	C–H bending
1431	0.59	27	1463	31	0.48	C–H bending
1552	0.10	30	1585	12	0.19	C=C stretching

[a]FVP in Ar matrix at 700 K. [b]Calculated at the B3LYP-D3/def2-TZVP level of theory. [c]Absolute intensities. [d]Relative intensities based on the strongest absorption.

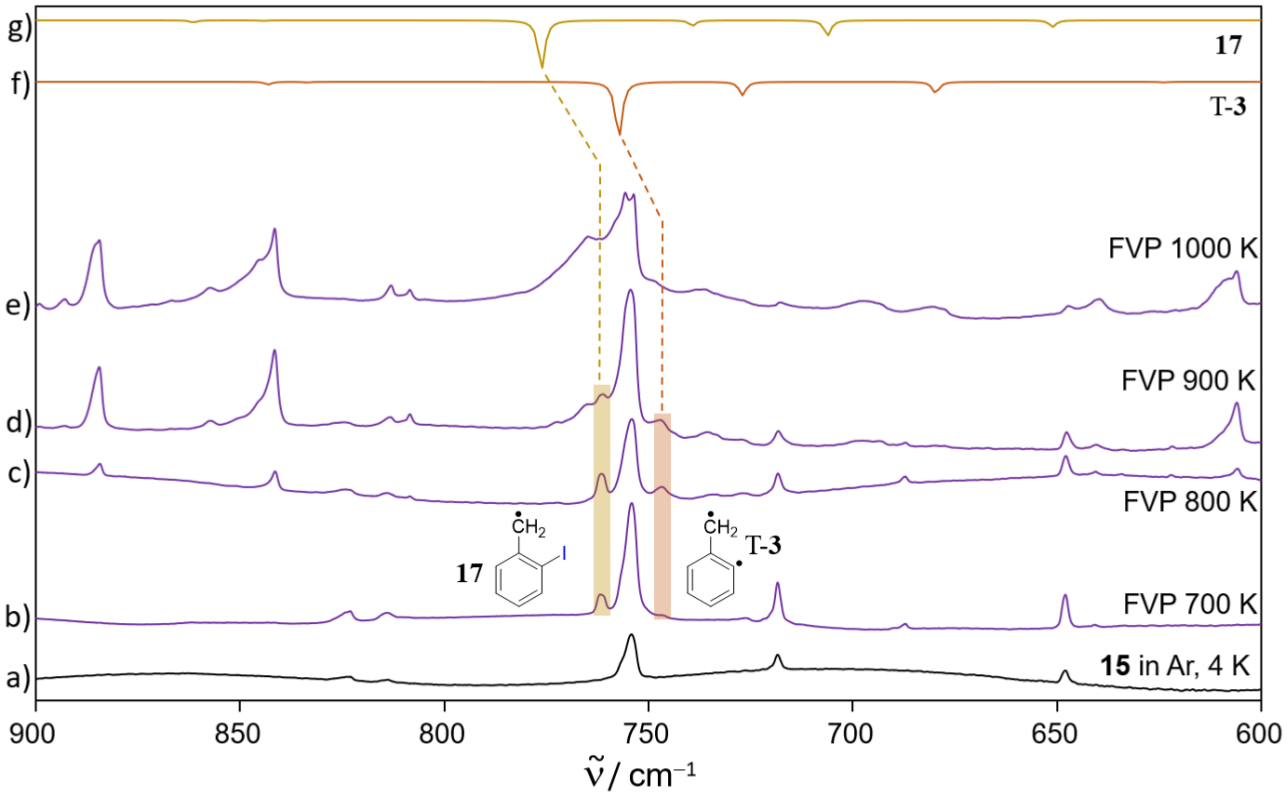

Figure S12. IR spectra of the FVP of 2-iodobenzyl iodide **15** in Ar matrices at 4 K. a) Deposition of **15** at RT (pyrolysis off). b-e) FVP of **15** at 700–1000 K. f-g) Calculated IR spectra of triplet diradical **T-3** and radical **17** at the B3LYP-D3/def2-TZVP level of theory.

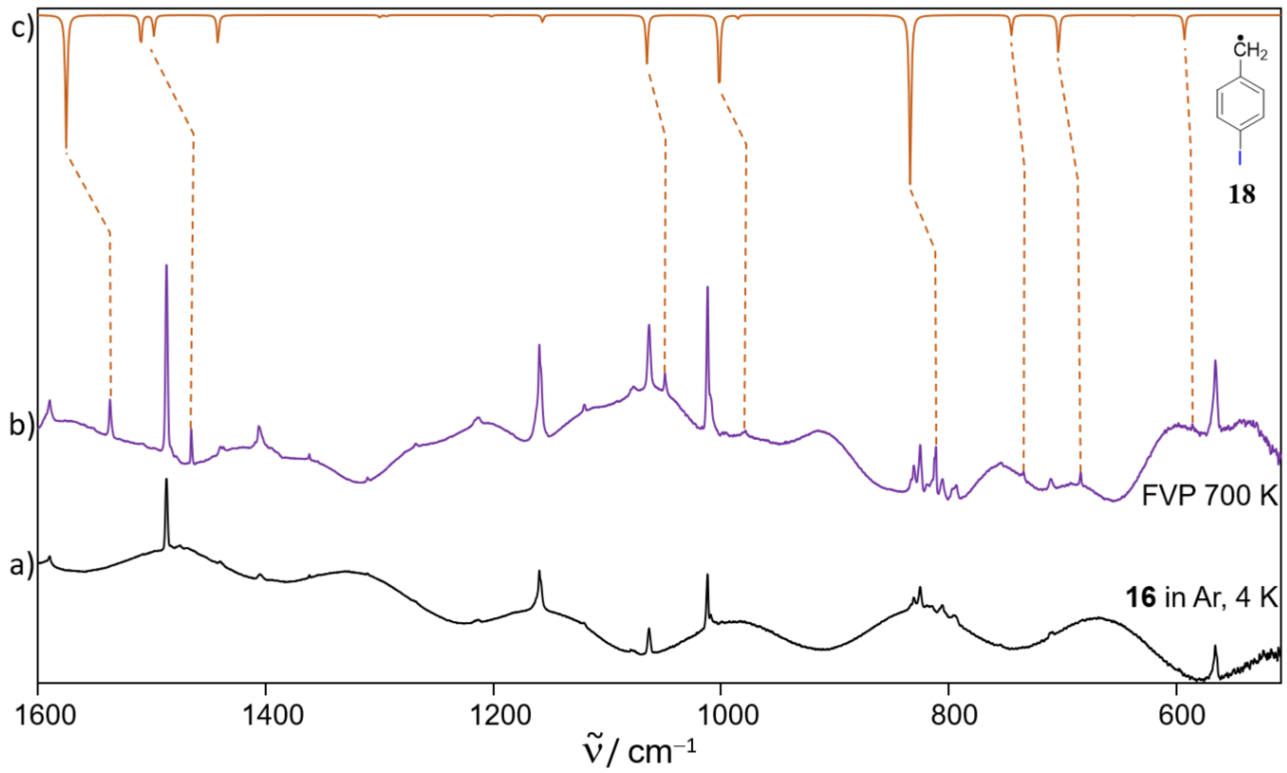

Figure S13. IR spectra of the FVP of 4-iodobenzyl iodide **16** in Ar matrices at 4 K. a) Deposition of **16** at RT (pyrolysis off). b) FVP of **16** at 700 K c) Calculated IR spectrum of radical **18** at the B3LYP-D3/def2-TZVP level of theory.

Table S3. Experimental and calculated IR spectroscopic data of 4-iodobenzyl radical 18.

Argon[a]		Calculated[b]				Assignment
ṽ (cm^{-1})	$I_{rel.}$[d]	normal mode	ṽ (cm^{-1})	$I_{abs.}$[c] (km/mol)	$I_{rel.}$[d]	
586	0.12	9	593	9	0.15	C–C–C bending
684	0.21	11	704	15	0.26	C–H wagging
734	0.21	12	745	8	0.13	C–H wagging
811	1.00	15	834	59	1.00	C–H wagging
979	0.20	19	1001	32	0.54	C–C–C bending
1049	0.38	20	1065	19	0.31	C–C–C bending
1406	0.15	26	1442	11	0.18	C–H bending
1465	0.17	28	1509	12	0.20	C–H bending (-CH_2)
1536	0.82	30	1575	51	0.86	C=C stretching (sym)

[a]FVP in Ar matrix at 700 K. [b]Calculated at the B3LYP-D3/def2-TZVP level of theory. [c]Absolute intensities. [d]Relative intensities based on the strongest absorption.

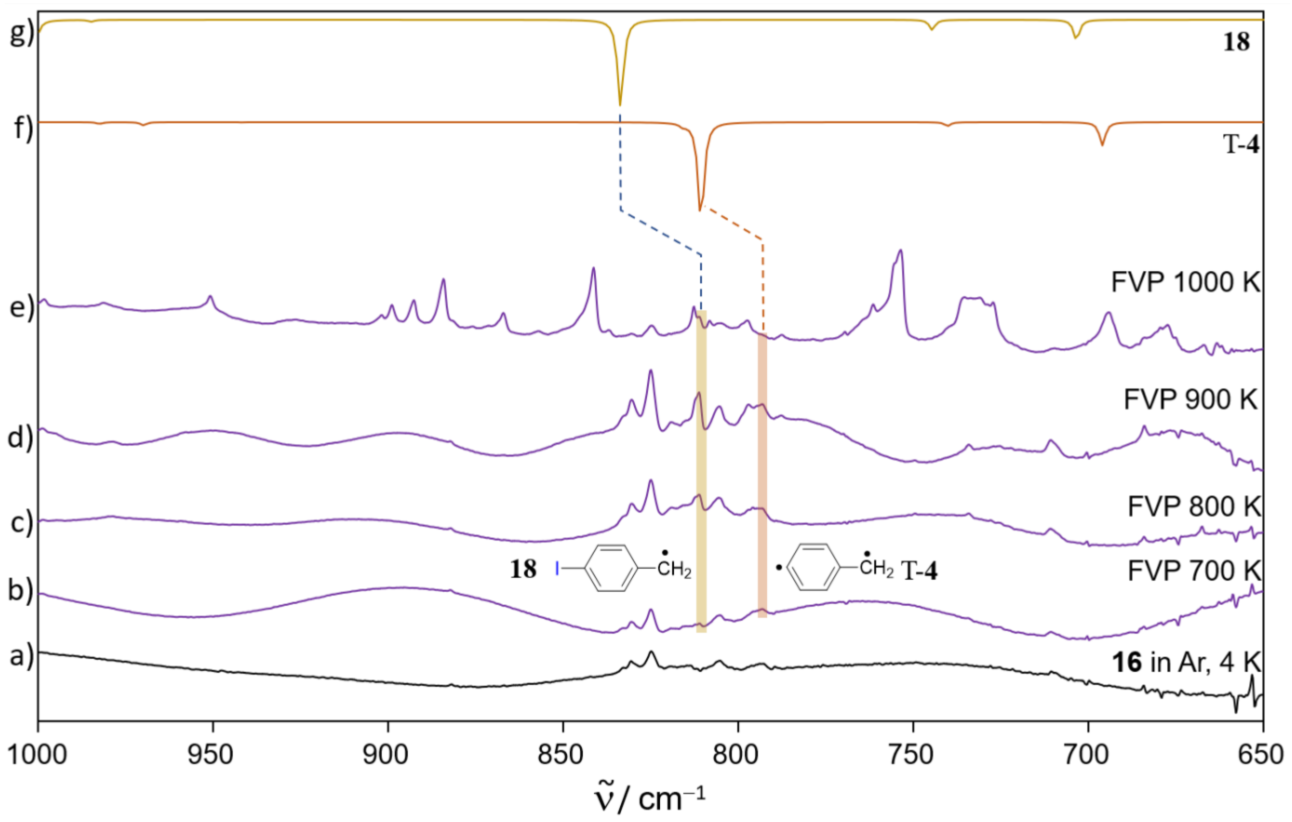

Figure S14. IR spectra of the FVP of 4-iodobenzyl iodide **16** in Ar matrices at 4 K. a) Deposition of **16** at RT (pyrolysis off). b-e) FVP of **16** at 700–1000 K. f-g) Calculated IR spectra of triplet diradical T-**4** and radical **18** at the B3LYP-D3/def2-TZVP level of theory.

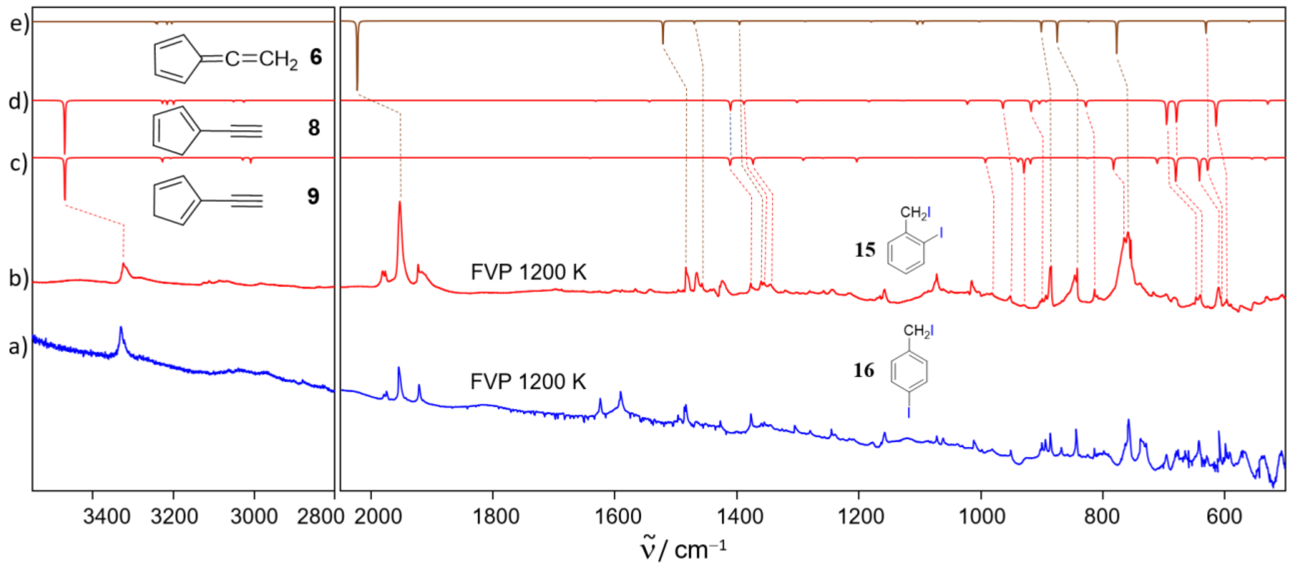

Figure S15. a-b) Comparison of the FVP of 2- and 4-iodobenzyl iodide at 1200 K. c-e) Calculated IR spectra of isomers **9**, **8**, and **6** at the B3LYP-D3/def2-TZVP level of theory.

Potential Energy Surface of C_7H_6

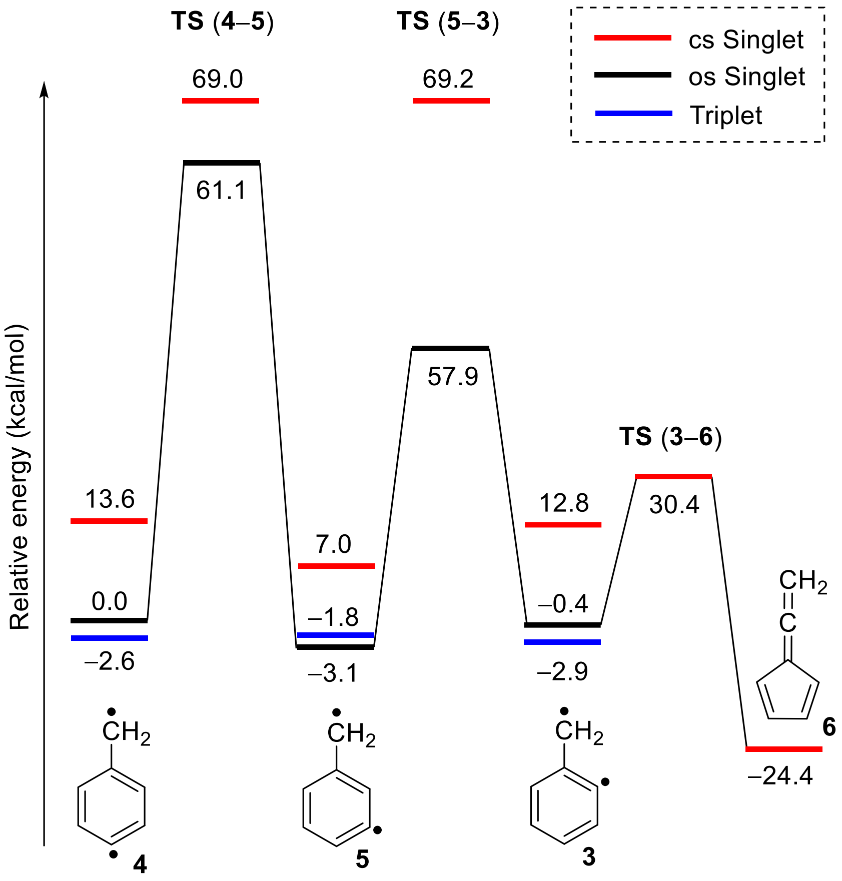

Figure S16. Potential energy surface for thermal rearrangement of diradicals **3**–**5** to fulvenallene **6** calculated at the ZPE-corrected B3LYP-D3/def2-TZVP level of theory. Closed-shell singlet (red), open-shell singlet (black) and triplet (blue) states and pathways are shown.

Optimized Geometries

Geometry optimizations at the B3LYP-D3/def2-TZVP level of theory in the gas phase.

**17**				**S-17$^+$**			
C	-0.17289490	0.51140320	0.00000000	C	-0.18235293	0.46522078	0.00000000
C	-1.42132509	1.10690069	0.00000000	C	-1.42633490	1.06889781	0.00000000
C	-1.54264386	2.49606601	0.00000000	C	-1.53554472	2.45949564	0.00000000
C	-0.39539486	3.29239764	0.00000000	C	-0.41022594	3.30480014	0.00000000
C	0.84905194	2.70568196	0.00000000	C	0.83142077	2.74426531	0.00000000
C	1.02648995	1.28842114	0.00000000	C	1.00646751	1.30848359	0.00000000
H	-2.31216702	0.49412254	0.00000000	H	-2.32566998	0.46938058	0.00000000
H	-2.52654976	2.94636598	0.00000000	H	-2.52628005	2.89855871	0.00000000
H	-0.48347280	4.37133608	0.00000000	H	-0.53750253	4.37828515	0.00000000
H	1.73867915	3.32374230	0.00000000	H	1.71966004	3.36330252	0.00000000
C	2.31985860	0.75907884	0.00000000	C	2.27683705	0.82118364	0.00000000
H	2.50626416	-0.30343382	0.00000000	H	2.49045136	-0.24011076	0.00000000
H	3.17341543	1.42232394	0.00000000	H	3.12515991	1.49471093	0.00000000
I	-0.11462301	-1.60781422	0.00000000	I	-0.10014000	-1.61128695	0.00000000
**T-17$^+$**				**18**			
C	-0.15409919	0.47696241	0.00000000	C	0.00000000	1.21222200	-2.28320412
C	-1.43872202	1.08659868	0.00000000	C	0.00000000	1.21338700	-0.90446912
C	-1.57005833	2.46221313	0.00000000	C	0.00000000	0.00000000	-0.21006712
C	-0.41292477	3.24220158	0.00000000	C	0.00000000	-1.21338700	-0.90446912
C	0.86648962	2.65359136	0.00000000	C	0.00000000	-1.21222200	-2.28320412
C	1.06028568	1.25827620	0.00000000	C	0.00000000	0.00000000	-3.02764812
H	-2.32119550	0.46079841	0.00000000	H	0.00000000	2.15023200	-0.36438612
H	-2.54919737	2.91924973	0.00000000	H	0.00000000	-2.15023200	-0.36438612
H	-0.48958502	4.32236009	0.00000000	H	0.00000000	-2.15534400	-2.81623712
H	1.73726595	3.29678592	0.00000000	C	0.00000000	0.00000000	-4.42577312
C	2.35868648	0.73421954	0.00000000	H	0.00000000	0.92584500	-4.98418112
H	2.55889106	-0.32598658	0.00000000	H	0.00000000	-0.92584500	-4.98418112
H	3.20742679	1.40256974	0.00000000	I	0.00000000	0.00000000	1.89740788
I	-0.12078398	-1.57660669	0.00000000	H	-0.00000000	2.15534400	-2.81623712
**S-18$^+$**				**T-18$^+$**			
C	0.00000000	-1.23613200	-2.25048289	C	0.00000000	-1.22565500	-2.25052073
C	0.00000000	-1.23404200	-0.88649589	C	0.00000000	-1.23595800	-0.87975973
C	0.00000000	0.00000000	-0.19346389	C	0.00000000	0.00000000	-0.17842073
C	-0.00000000	1.23404200	-0.88649589	C	-0.00000000	1.23595800	-0.87975973
C	-0.00000000	1.23613200	-2.25048289	C	-0.00000000	1.22565500	-2.25052073
C	0.00000000	0.00000000	-2.98609289	C	0.00000000	0.00000000	-2.97529873
H	0.00000000	-2.16331100	-0.33462389	H	0.00000000	-2.17168700	-0.33795573
H	-0.00000000	2.16331100	-0.33462389	H	-0.00000000	2.17168700	-0.33795573
H	0.00000000	2.17220700	-2.79494389	H	0.00000000	2.16179600	-2.79303773
C	0.00000000	0.00000000	-4.34834289	C	0.00000000	0.00000000	-4.40537173
H	0.00000000	-0.92436100	-4.91351889	H	0.00000000	-0.92866400	-4.95776973
H	-0.00000000	0.92436100	-4.91351889	H	-0.00000000	0.92866400	-4.95776973
I	0.00000000	0.00000000	1.86598711	I	0.00000000	0.00000000	1.86972527
H	0.00000000	-2.17220700	-2.79494389	H	0.00000000	-2.16179600	-2.79303773

**OSS-3**				**T-3**			
C	-1.20911719	0.19387153	0.00000000	C	1.21175945	0.19321266	0.00000000
C	-1.23027050	-1.16245719	0.00000000	C	0.08276616	1.02670531	0.00000000
C	0.01558257	-1.82332158	0.00000000	C	-1.15961694	0.31203214	0.00000000
C	1.19104335	-1.07336633	0.00000000	C	-1.19440796	-1.06684692	0.00000000
C	1.15681835	0.31023349	0.00000000	C	-0.01527876	-1.82267198	0.00000000
C	-0.07914287	1.02434209	0.00000000	C	1.22775273	-1.16835407	0.00000000
H	2.14740639	-1.58108985	0.00000000	H	-2.08125566	0.88314490	0.00000000
H	2.07983472	0.87935998	0.00000000	H	-2.15071235	-1.57479783	0.00000000
C	-0.14319913	2.42656527	0.00000000	C	0.14650639	2.42235545	0.00000000
H	-1.09432110	2.93790427	0.00000000	H	1.09722291	2.93508782	0.00000000
H	0.76050617	3.01995026	0.00000000	H	-0.75687321	3.01645538	0.00000000
H	0.05198355	-2.90549713	0.00000000	H	-0.05710477	-2.90463769	0.00000000
H	-2.15569716	-1.72583125	0.00000000	H	2.15183683	-1.73384816	0.00000000
**OSS-4**				**T-4**			
C	0.00000000	1.21709000	0.18661412	C	0.00000000	1.22072600	0.18576904
C	0.00000000	1.22290800	-1.20392688	C	0.00000000	0.00000000	0.92250004
C	0.00000000	0.00000000	-1.83068388	C	0.00000000	-1.22072600	0.18576904
C	0.00000000	-1.22290800	-1.20392688	C	0.00000000	-1.22499600	-1.20039896
C	0.00000000	-1.21709000	0.18661412	C	0.00000000	0.00000000	-1.83284296
C	0.00000000	0.00000000	0.91867512	C	-0.00000000	1.22499600	-1.20039896
H	0.00000000	2.15536700	-1.75467188	H	0.00000000	2.15971900	0.72732104
H	0.00000000	-2.15536700	-1.75467188	H	0.00000000	-2.15971900	0.72732104
H	0.00000000	-2.15676600	0.72708612	H	0.00000000	-2.15727000	-1.75162396
C	0.00000000	0.00000000	2.32745212	H	-0.00000000	2.15727000	-1.75162396
H	0.00000000	0.92604600	2.88513212	C	0.00000000	0.00000000	2.32107704
H	0.00000000	-0.92604600	2.88513212	H	0.00000000	0.92561900	2.87988004
H	-0.00000000	2.15676600	0.72708612	H	0.00000000	-0.92561900	2.87988004
**OSS-5**				**T-5**			
C	-1.25865801	0.32077775	0.00000000	C	-1.25794194	0.32011489	0.00000000
C	-1.28425733	-1.03766663	0.00000000	C	0.02353630	0.96120678	0.00000000
C	-0.19497812	-1.88063914	0.00000000	C	1.17158690	0.11960855	0.00000000
C	1.06643113	-1.25707563	0.00000000	C	1.06595713	-1.25692723	0.00000000
C	1.17287408	0.11893409	0.00000000	C	-0.19433927	-1.88026932	0.00000000
C	0.02370162	0.96308050	0.00000000	C	-1.28310515	-1.03781505	0.00000000
H	1.96138050	-1.86816489	0.00000000	H	-2.15957620	0.92120004	0.00000000
H	2.15222281	0.58161639	0.00000000	H	2.15079061	0.58254941	0.00000000
C	0.13647163	2.35567957	0.00000000	H	1.96116166	-1.86768590	0.00000000
H	-0.74076113	2.98775230	0.00000000	C	0.13618350	2.35683350	0.00000000
H	1.10519945	2.83642064	0.00000000	H	-0.74125851	2.98847562	0.00000000
H	-2.15994299	0.92265309	0.00000000	H	1.10496195	2.83725281	0.00000000
H	-0.28760847	-2.95882054	0.00000000	H	-0.28734429	-2.95830469	0.00000000

TS$_{(4-5)}$				TS$_{(5-3)}$			
C	-0.11148721	1.18732600	0.00750509	C	-0.23936511	-1.22329597	-0.15057101
C	1.26476379	1.15600905	0.04860912	C	-1.02714694	-0.02302286	-0.03527298
C	1.82762184	-0.12690193	-0.11967792	C	-0.21524878	1.15037103	-0.06885701
C	1.08748888	-1.23640196	-0.08207498	C	1.17892921	1.10859884	0.00994793
C	-0.29452112	-1.27206401	0.05807398	C	1.88636705	-0.08880725	0.08787290
C	-0.92942017	0.00110497	-0.00436998	C	1.08337989	-1.24196715	-0.08785806
H	1.85122975	2.05938206	0.14936317	H	0.39134279	-2.15343405	0.54045497
H	2.31174687	-1.24180193	0.40962205	H	-0.70821266	2.11453710	-0.13062400
H	-0.87470709	-2.17524404	0.19376693	H	1.72308534	2.04657377	0.02505791
C	-2.32745417	0.10930891	-0.02524201	C	-2.40762893	0.00027433	0.08612108
H	-2.95387414	-0.77186711	-0.02102207	H	-2.97572106	-0.91863360	0.08258010
H	-2.81426921	1.07482990	-0.03462399	H	-2.94264780	0.93418940	0.19622410
H	-0.62207725	2.14441498	0.00595411	H	2.95643506	-0.11613840	0.23800986
TS$_{(3-6)}$							
C	-0.16006997	-0.98314393	0.19997188				
C	1.14230607	-1.21246878	0.08048604				
C	1.91528895	-0.01188169	-0.12737093				
C	1.15092781	1.12253122	-0.08401907				
C	-0.23741321	1.02647306	0.18262278				
C	-1.17804904	-0.09479706	-0.06306527				
H	1.55905121	-2.20257773	-0.05941187				
H	2.95295599	-0.04710258	-0.43054782				
H	1.59809170	2.10859027	-0.12939107				
H	-0.65785437	1.76737305	0.85855570				
C	-2.51904703	-0.09171923	-0.17077742				
H	-3.04421712	0.83084170	-0.36865052				
H	-3.09168895	-0.98708629	0.02235756				

References

1. Hemberger, P.; Wu, X. K.; Pan, Z. Y.; Bodi, A., Continuous Pyrolysis Microreactors: Hot Sources with Little Cooling? New Insights Utilizing Cation Velocity Map Imaging and Threshold Photoelectron Spectroscopy. *J Phys Chem A* **2022,** *126* (14), 2196-2210.
